# LTR retroelement expansion of the human cancer transcriptome and immunopeptidome revealed by de novo transcript assembly

**DOI:** 10.1101/gr.248922.119

**Published:** 2019-10

**Authors:** Jan Attig, George R. Young, Louise Hosie, David Perkins, Vesela Encheva-Yokoya, Jonathan P. Stoye, Ambrosius P. Snijders, Nicola Ternette, George Kassiotis

**Affiliations:** 1 Retroviral Immunology, The Francis Crick Institute, London NW1 1AT, United Kingdom;; 2 Retrovirus-Host Interactions, The Francis Crick Institute, London NW1 1AT, United Kingdom;; 3 Mass Spectrometry Proteomics, The Francis Crick Institute, London NW1 1AT, United Kingdom;; 4 Department of Medicine, Faculty of Medicine, Imperial College, London W2 1PG, United Kingdom;; 5 The Jenner Institute, University of Oxford, Oxford OX3 7DQ, United Kingdom

## Abstract

Dysregulated endogenous retroelements (EREs) are increasingly implicated in the initiation, progression, and immune surveillance of human cancer. However, incomplete knowledge of ERE activity limits mechanistic studies. By using pan-cancer de novo transcript assembly, we uncover the extent and complexity of ERE transcription. The current assembly doubled the number of previously annotated transcripts overlapping with long-terminal repeat (LTR) elements, several thousand of which were expressed specifically in one or a few related cancer types. Exemplified in melanoma, LTR-overlapping transcripts were highly predictable, disease prognostic, and closely linked with molecularly defined subtypes. They further showed the potential to affect disease-relevant genes, as well as produce novel cancer-specific antigenic peptides. This extended view of LTR elements provides the framework for functional validation of affected genes and targets for cancer immunotherapy.

The human genome hosts diverse families of endogenous retroelements (EREs), many of which have amplified their copies to staggering numbers (International Human Genome Sequencing Consortium 2001; de Koning et al. 2011). These comprise human endogenous retroviruses (HERVs) and mammalian apparent long-terminal repeat (LTR)-retrotransposons (MaLRs), distinguished by LTRs flanking the canonical proviral genomes and collectively referred to as LTR elements, and the larger group of non-LTR elements, which include long and short interspersed nuclear elements (LINEs and SINEs, respectively) and composite SINE-VNTR-*Alu* (SVA) elements (Burns and Boeke 2012; Feschotte and Gilbert 2012). The vast majority of human genomic ERE integrations are incomplete and mutated copies, incapable of replication. Nevertheless, many retain functional parts, including promoter or enhancer activities of the LTRs, splice donor and acceptor sites, polyadenylation sites, and even intact open reading frames (ORFs) for biologically active proteins, all of which have the potential to alter host physiology (Burns and Boeke 2012; Feschotte and Gilbert 2012; Kassiotis and Stoye 2016). Potential risks posed by functional components of ERE integrations are mitigated by epigenetic and splicing repression, largely preventing ERE expression or exonization (Feschotte and Gilbert 2012). However, this type of epigenetic control is reversible and often lost, particularly in the context of the altered chromatin landscape of cancer initiation and evolution (Baylin and Jones 2011; Flavahan et al. 2017).

Dysregulated EREs can affect cancer progression through distinct mechanisms (Romanish et al. 2010; Kassiotis and Stoye 2017). Examples include LTR-driven overexpression of proto-oncogenes, such as *CSF1R* overexpression in Hodgkin's lymphoma and anaplastic large-cell lymphoma (Lamprecht et al. 2010), or creation of truncated oncogenic forms of kinases through alternative splicing to an LTR element, such as anaplastic lymphoma kinase (ALK) in melanoma (Wiesner et al. 2015), and the erb-b2 receptor tyrosine kinase 4 (ERBB4) in ALK-negative anaplastic large-cell lymphoma (Scarfo et al. 2016). Genetic, pharmacologic, or cytokine-mediated transcriptional induction of LTR elements is incriminated in the activation of cancer cell–intrinsic innate immunity (Chiappinelli et al. 2015; Roulois et al. 2015; Goel et al. 2017; Cañadas et al. 2018; Sheng et al. 2018), and expression of LTR element clusters is linked with the strength of local antitumor immunity and the outcome of immunotherapy (Rooney et al. 2015; Desai et al. 2017; Smith et al. 2018). Moreover, canonical retroviral proteins or protein fragments encoded by a few distinct HERV-K, HERV-E, and HERV-H proviruses can be targeted by functionally relevant T cell and B cell responses (Schiavetti et al. 2002; Rakoff-Nahoum et al. 2006; Takahashi et al. 2008; Mullins and Linnebacher 2012; Cherkasova et al. 2016; Smith et al. 2018), but the potential of the multitude of noncanonical or chimeric ERE transcripts to generate cancer-specific antigenic epitopes has not yet been explored.

Despite their potential importance, complete understanding of the role of EREs in cancer is currently hampered by gaps in the annotation and quantitation of the transcriptional activity in cancer of the compendium of ERE-overlapping transcripts the human genome can produce. By using de novo assembly of cancer transcriptomes, we aimed to provide a comprehensive view of LTR element transcriptional behavior in human cancer.

## Results

### A comprehensive LTR retroelement transcriptome

**Figure 1.**
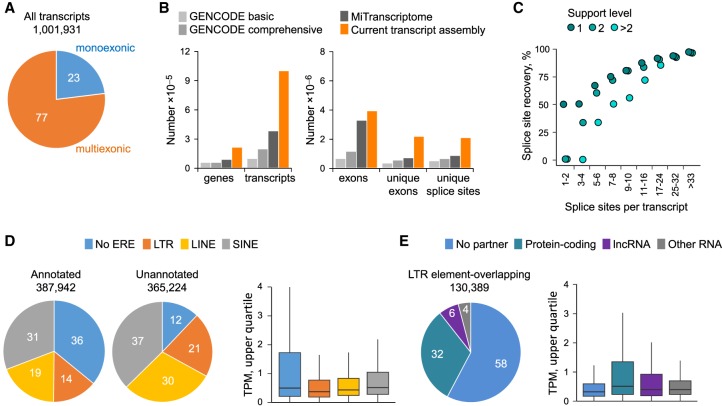
Assembly, recovery, and expression of ERE-overlapping transcripts in tumors of diverse origins. (*A*) Total number and proportion of monoexonic or multiexonic de novo–assembled transcripts. (*B*) Comparison of the total number of genes, transcripts, exons, unique exons, and unique splice sites in the current transcript assembly with GENCODE (version 24) (Frankish et al. 2019) and MiTranscriptome (Iyer et al. 2015). Genes are defined here as nonoverlapping transcribed regions. (*C*) Completeness of the current transcript assembly, estimated by median recovery of splice sites annotated in GENCODE. The percentage of GENCODE recovered sites is plotted according to their support levels. Recovery of the 367,411 unique splice sites of high-confidence GENCODE transcripts was ∼93%. (*D*) Prior annotation status and ERE composition of the 753,166 transcripts out of the entire assembly that were expressed at one or more transcripts per million (TPM) in at least one sample (*left*) and expression levels of these transcripts according to their ERE composition (*right*). Transcripts were considered as previously annotated if all exons were present within GENCODE (v24 basic) and as ERE-overlapping if any exon overlapped with an ERE integration. For transcripts overlapping with multiple EREs, we assigned a hierarchical LTR, LINE, or SINE order. As overall expression level, we used the upper quartile TPM in the cancer type with highest expression for each transcript. (*E*) Breakdown of LTR element–overlapping transcripts (expressed at one or more TPM in at least one sample) according to overlap with protein-coding, lncRNA, or other RNA genes (*left*) and expression levels (upper quartile TPM in the cancer type with highest expression) or each type of LTR element–overlapping transcript (*right*).

To identify and quantify unannotated or partially annotated LTR element–overlapping transcripts, we first de novo-assembled a comprehensive cancer transcriptome (Methods). To this end, RNA-seq reads from 768 patient samples, obtained from The Cancer Genome Atlas (TCGA) program and representing 31 cancer types (Supplemental Table S1), were used for genome-guided assembly. This process generated 1,001,931 contigs, the majority of which were multiexonic (Fig. 1A). Comparison with other comprehensive ab initio transcript assemblies, such as the Encyclopedia of DNA Elements subproject GENCODE (Frankish et al. 2019) and MiTranscriptome (Iyer et al. 2015), indicated considerably increased representation of genes, transcripts, unique exons, and unique splice sites in the current assembly (Fig. 1B). Moreover, comparison with splice sites in high-confidence GENCODE transcripts revealed recovery of 93% of all splice sites and an average of only one splice site missing per annotated transcript (Fig. 1C).

To examine the representation of LTR or non-LTR elements in the cancer transcriptome, the assembled contigs were overlaid with a genomic repeat sequence annotation, generated through RepeatMasker (Smit et al. 2013–2015). We first concentrated on transcripts with expression level of one or more transcripts per million (TPM) in at least one sample (a total of 753,166 transcripts). These comprised both previously annotated (fully or partially) and unannotated transcripts in similar proportions (Fig. 1D). Transcripts overlapping EREs, particularly LTR elements or LINEs, were considerably enriched in the unannotated fraction (Fig. 1D). Expression of such transcripts was, on average, lower than of transcripts that did not include any EREs (Fig. 1D). Hence, the assembly captured transcripts using EREs, including those expressed at comparably lower levels.

Closer inspection of the subset of transcripts that contained at least one complete or partial LTR element revealed that more than half did not intersect any annotated gene (Fig. 1E). Approximately one-third of LTR element–overlapping transcripts spanned a protein-coding gene, whereas a much smaller proportion spanned long noncoding RNA (lncRNA) or other RNA genes (Fig. 1E). Expression of LTR element–overlapping transcripts was significantly higher if they also spanned protein-coding genes or lncRNA genes than if they were stand-alone LTR elements (Fig. 1E). These findings suggest that the current assembly was enriched for splice isoforms of annotated genes, as well as previously unannotated ERE-overlapping transcripts, likely owing to lack of bias against repetitive elements, compared with previous assemblies (Iyer et al. 2015; Frankish et al. 2019).

### Cancer-specific LTR retroelement–overlapping transcripts

As the transcript assembly was based on cancer sample RNA-seq reads, it was expected to include transcripts that were expressed in a cancer-specific manner. To identify such cancer-specific LTR element–overlapping transcripts (referred to here as CLTs), we compared cancer samples with a collection of 811 samples from a wide variety of healthy tissues, using RNA-seq data obtained from TCGA, as well as the Genotype Tissue Expression (GTEx) Consortium (Supplemental Tables S2, S3). Transcripts were considered cancer-specific if they fulfilled the following criteria: (1) Their 75th percentile expression in a given cancer type was more than one TPM, three or more times the highest median expression in any healthy tissue, and three or more times the 90th percentile expression in the respective healthy tissue, and (2) their 90th percentile expression in healthy tissue samples was less than 10 TPM. This combination pruned the number of potential transcripts down to a total of 5923 transcripts containing complete or partial LTR elements and expressed in a cancer-specific manner (Supplemental Table S4).

As further validation, we intersected the identified CLTs with previously described examples of LTR element exaptation in the expression of oncogenes and cancer-associated lncRNAs (Babaian and Mager 2016; Jang et al. 2019). Although 73% (92 of 116) of precompiled loci (Babaian and Mager 2016; Jang et al. 2019) were present in the assembly, only 15 of them fulfilled the cancer specificity criteria we set (Supplemental Table S5), with the remaining either expressed also in healthy tissue or not expressed sufficiently recurrently in cancer patients.

Although a substantial number, the identified CLTs represented only a small proportion of genomic or transcriptionally used LTR elements. For example, of a total of 630,356 annotated genomic LTR elements, 108,946 (17.3%) were present in assembled transcripts and expressed at one or more TPM in at least one healthy or tumor sample. Of those, only 20,164 (3.2% of annotated LTR elements) were expressed specifically in cancer, with the remaining expressed either additionally (12.8%) or only (1.3%) in healthy tissues. When considering recurrence of expression between individuals, the 5923 CLTs expressed specifically and recurrently in cancer overlap with only 0.9% of annotated LTR elements, contrasting with a total of 66,277 LTR-overlapping transcripts expressed recurrently in healthy tissues (using 8.5% of annotated LTR elements). Therefore, LTR elements used specifically and recurrently in cancer represent one in 10 LTR elements used in healthy tissues and one in 100 genomic LTR elements.

**Figure 2.**
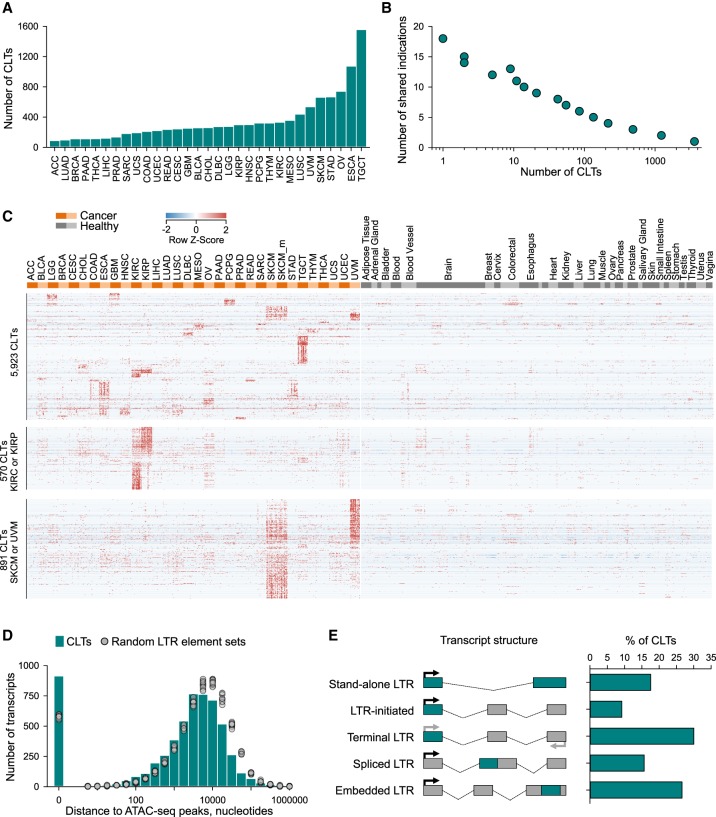
Abundance of cancer-specific LTR element–overlapping transcripts. (*A*) Total number of CLTs identified per cancer type. (*B*) Overlap of CLT expression between cancer types, plotted as the number of CLTs against the number of cancer types sharing a given CLT. (*C*) Heatmap of expression values in cancer patient and healthy control samples of all 5923 identified CLTs (*top*), KIRC-specific and KIRP-specific CLTs (570) (*middle*), and SKCM-specific and UVM-specific CLTs (891) (*bottom*). (*D*) Proximity, in nucleotides, of the identified CLT TSS to ATAC-seq peaks. Also shown is the proximity of ATAC-seq peaks to the center of 10 random sets of similar numbers of LTR elements. (*E*) Composition of identified CLTs according to the indicated position of the LTR element in the transcript structure.

Whereas most cancer types showed significantly elevated expression of 100–300 of the identified 5923 transcripts, a few stood out, with testicular germ cell tumors (TGCTs) and esophageal carcinoma (ESCA) each expressing more than 1000 such CLTs (Fig. 2A). Conversely, the vast majority (>95%) of CLTs were expressed specifically in one or up to five different cancer types, and only 44 CLTs were shared by 10 or more cancer types (Fig. 2B), showing tissue-type specificity of LTR element utilization. Indeed, although cancer-specific, expression of CLTs clustered primarily according to tissue type, with most clusters restricted to one cancer type (Fig. 2C). Consequently, a considerable degree of overlap in CLT expression was observed in cancer types involving related tissues, such as kidney renal clear cell carcinoma (KIRC) and kidney renal papillary cell carcinoma (KIRP) or histotypes such as skin cutaneous melanoma (SKCM), both primary and metastatic, and uveal melanoma (UVM) (Fig. 2C). Tissue-restricted expression of CLTs was further supported by analysis of RNA-seq data from 933 cancer cell lines from the Cancer Cell Line Encyclopedia (CCLE) (Supplemental Fig. S1), which are homogenous cell populations.

The distinct pattern of CLT expression among cancer types was suggestive of locus-specific epigenetic changes controlling LTR element activity. Indeed, transcription start sites (TSSs) of CLTs were in close proximity (*P* < 0.01) to assay for transposase-accessible chromatin using sequencing (ATAC-seq) peaks, identified in a recent mapping of enhancers and promoters in primary tumor samples (Fig. 2D; Corces et al. 2018), consistent with cancer specificity of CLT expression owing to activation of a local regulatory element. Consequently, transcriptional inclusion of LTR elements covered diverse LTR element families in similar proportions in each cancer type, with a few exceptions (Supplemental Fig. S2). These included prominent HERV-H element cluster in TCGTs and a few additional indications, such as colon adenocarcinoma (COAD) (Supplemental Fig. S2), consistent with prior reports (Pérot et al. 2015; Desai et al. 2017). They also included a HERV-K element cluster in prostate adenocarcinoma (PRAD) (Supplemental Fig. S2), consisting of both HERV-K (HML-2) and evolutionary older HERV-K elements in equal proportions (12 and 11 transcripts, respectively). Of note, the majority of the HERV-K (HML-2)–overlapping transcripts in PRAD derived from a single provirus on Chromosome 22q11.23 (*HERVK*[*Chr22q11.23*]), consistent with reported expression of this provirus in PRAD (Goering et al. 2015), and partially overlapped with recently identified lncRNA prostate cancer associated transcript 14 (*PCAT14*) (Shukla et al. 2016), indicating that *PCAT14* is, in fact, a *HERVK*[*Chr22q11.23*] transcript (Supplemental Fig. S2).

We next examined whether CLT expression resulted from derepression of LTR element promoter activity or from alternative promoters, by mapping the position of the LTR element in the overall structure of the identified CLTs. Stand-alone LTR elements represented 17.2% of CLTs, whereas an LTR element was found at the TSS in 9% of the CLTs (LTR-initiated) (Fig. 2E), suggesting it acted as the promoter of those transcripts. A terminal LTR element was found in a further 30% of the transcripts, whose strand was not known (Fig. 2E), although it is likely that the LTR element is at the TSS of the majority of these transcripts. In a quarter of cases, an LTR element was embedded in one of the exons, typically the last exon with an extended untranslated region (UTR) (embedded LTR), and in 15% of CLTs, an LTR element provided at least one splice site (spliced LTR) (Fig. 2E). The latter transcripts were chimeric, encompassing an LTR element and either a protein-coding or lncRNA gene in equal proportions (48% and 52%, respectively).

**Figure 3.**
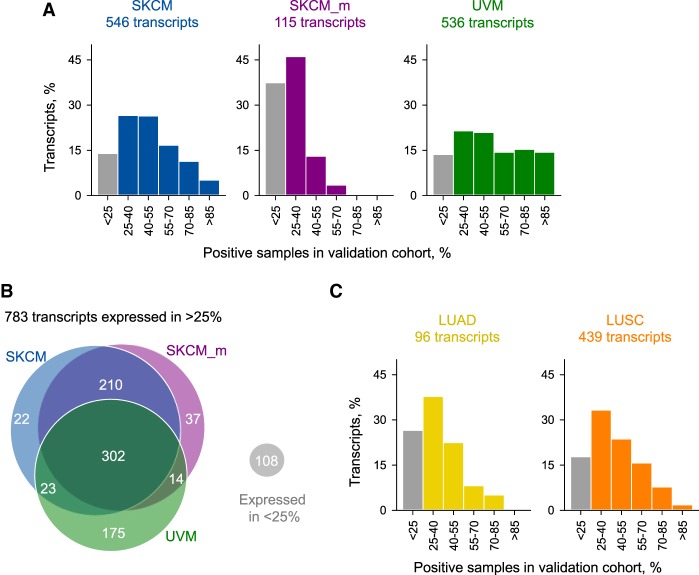
Validation of CLT expression prevalence. (*A*) Percentage of CLTs expressed in each bin of percentage of positive samples in larger cohorts of primary SKCM (*n* = 77), metastatic SKCM (SKCM_m; *n* = 318), or UVM (*n* = 31). (*B*) Number and overlap between melanoma types of CLTs that were validated in the larger cohorts, that is, expressed in >25% of cancer patient samples in the validation cohort. (*C*) Percentage of CLTs expressed in each bin of percentage of positive samples in larger cohorts of LUAD (*n* = 395) or LUSC (*n* = 338). Samples were considered positive if transcript expression level was more than three times that of the highest median in any normal tissue.

The structures of CLTs, as well as the specificity of their expression to individual cancer types, suggested a high degree of predictability of LTR element utilization through mechanisms, including LTR element–initiated transcription, as well as alternative splicing and inclusion of cryptic exons in transcripts initiated by alternative promoters. To confirm cancer specificity and prevalence of CLT expression, we extended our analysis to a larger cohort. For this purpose, we selected SKCM as an indication with well-characterized publicly available data and UVM as a related cancer histotype and analyzed a further 77 primary and 318 metastatic SKCM samples and 31 UVM samples. Of the 546 CLTs selected based on expression in more than a quarter of the primary SKCM discovery cohort, 470 (86%) were also expressed in more than a quarter of the primary SKCM validation cohort (Fig. 3A). In fact, primary SKCM-specific LTR element–overlapping transcripts were expressed on average in 60%, with some expressed in 98% of the analyzed samples (Fig. 3A). Highly comparable results were obtained also with the UVM validation cohort, in which 86% (463 of 536) of UVM-specific LTR element–overlapping transcripts were expressed above the threshold (Fig. 3A). Although still the majority (62%), only 72 of 115 CLTs expressed in the metastatic SKCM discovery cohort were expressed above the threshold also in the metastatic SKCM validation cohort (Fig. 3A). Moreover, in contrast to primary SKCM and UVM samples, only four CLTs were expressed in more than half and none were expressed in >70% of metastatic SKCM samples (Fig. 3A). Nevertheless, more than half (57%) of CLTs expressed in primary SKCM were also expressed in metastatic SKCM, and more than one-third (34%) were shared between SKCM and UVM (Fig. 3B). Similar validation rates were also obtained from analysis of further 395 lung adenocarcinoma (LUAD) and 338 lung squamous cell carcinoma (LUSC) samples, with 73% and 82% of CLTs identified based on expression in more than a quarter of each discovery cohort, also expressed in more than a quarter of the LUAD and LUSC validation cohorts, respectively (Fig. 3C). Validation rates in these cancer types were also comparable between LTR-initiated and other CLTs (Supplemental Fig. S3), indicating that LTR promoters can be used as recurrently between cancer patients as canonical promoters, in agreement with a recent independent study (Jang et al. 2019). Together, these results highlighted the abundance of LTR element–overlapping transcripts expressed specifically and predictably in cancer.

### Individual CLT expression patterns associated with melanoma subtypes and progression rate

**Figure 4.**
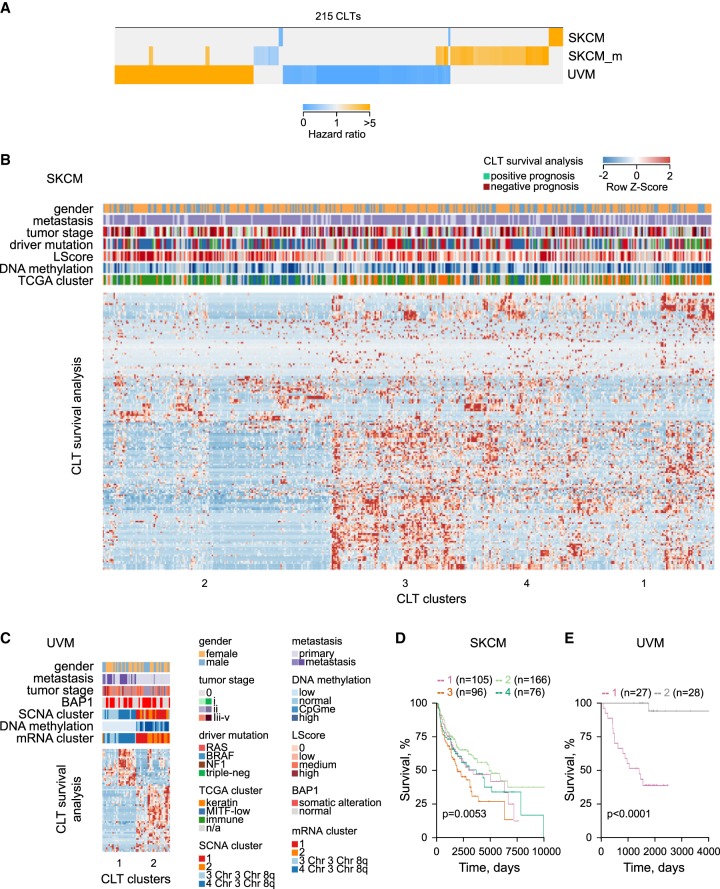
Potential biological processes underlying melanoma association of melanoma-expressed CLTs. (*A*) Heatmap of hazard ratios, calculated by Cox regression model, of the 215 melanoma-expressed CLTs that were significantly associated with survival probability for each melanoma type of patients in the higher versus the lower expression tertiles. (*B*,*C*) Unsupervised clustering of 180 SKCM-prognostic CLTs (*B*) and 67 UVM-prognostic CLTs (*C*), according to their expression values (*x*-axis) and effect on survival probability (*y*-axis). Also annotated are TCGA-defined clinical and molecular subtypes: (LScore) lymphocyte infiltration score (The Cancer Genome Atlas Network 2015; Robertson et al. 2017). (*D*,*E*) Kaplan–Meier plots and *P*-values from Cox multi-regression model for patients stratified according to the four CLT clusters identified in SKCM (*D*) and the CLT two clusters identified in UVM (*E*).

To further probe underlying reasons for cancer specificity of CLT expression, we investigated if expression of different CLTs associated with distinct cancer stages or subtypes. To this end, we focused on melanoma and looked for correlation between CLT expression with rates of melanoma progression or clinical and molecular subtypes. Of the 891 CLTs expressed in either SKCM or UVM, 215 (24%) were significantly associated with altered disease progression, revealed by hazard ratios for death between the 33rd and 66th expression percentiles (Fig. 4A). This association appeared to be disease specific, with expression levels of only few CLTs linked with the same outcome in primary or metastatic SKCM and UVM (Fig. 4A).

Expression of prognostic CLTs in SKCM and UMV was independent from most clinical and genomic subtypes and known driver mutations but correlated well with molecular subclasses, previously identified by in-depth genomic and transcriptomic analyses (Fig. 4B–E; The Cancer Genome Atlas Network 2015; Robertson et al. 2017). Indeed, expression of CLTs associated with SKCM survival formed four distinct clusters, three of which matched clusters identified by TCGA (Fig. 4B,D). The cluster enriched in CLTs associated with the best prognosis (cluster 2) was enriched in the “immune” and “MITF-low” molecular signatures, whereas the cluster enriched in CLTs associated with worst prognosis (cluster 3) was enriched in the “keratin” signature (Fig. 4B). Similarly, expression of CLTs associated with UVM survival defined two clearly distinguishable and highly prognostic clusters (Fig. 4C,E). The cluster comprising all CLTs associated with better prognosis of UVM (cluster 2) was linked with low metastatic potential, Chr 3 and Chr 8q disomy, and preservations of normal DNA methylation patterns (Fig. 4C).

These results suggested that distinct and likely biologically relevant forces drive expression of CLT clusters in the different SKCM and UVM subtypes, a notion that was supported by detailed analysis of individual prognostic CLTs. One such example is the [*TRPM1*]*MLT1A0* transcript, a truncated variant of *TRPM1*, which encodes melastatin (Supplemental Fig. S4). The [*TRPM1*]*MLT1A0* transcript corresponds to *TRPM1 variant 4* (*TRPM1-210*; NM_001252030.1), with the exception of an extended 3′ UTR, created by exonization of an intronic *MLT1A0* LTR element, which serves as a terminal exon, replacing the last 24 exons of the canonical *TRPM1* transcript (Supplemental Fig. S4). *TRPM1* is a target of MITF, expressed in healthy, as well as transformed cutaneous and uveal melanocytes (Duncan et al. 1998; Miller et al. 2004), whereas [*TRPM1*]*MLT1A0* was found principally expressed in SKCM and UVM (Supplemental Fig. S4). Levels of [*TRPM1*]*MLT1A0* were directly proportional to those of *TRPM1* in SKCM, and their down-regulation in a fraction of metastatic SKCM patients (Duncan et al. 1998) was linked with improved survival probability (Supplemental Fig. S4), as would expected for a MITF target (The Cancer Genome Atlas Network 2015). In UVM samples, however, higher expression of [*TRPM1*]*MLT1A0* was at the expense of *TRPM1* expression and linked with better prognosis (Supplemental Fig. S4).

**Figure 5.**
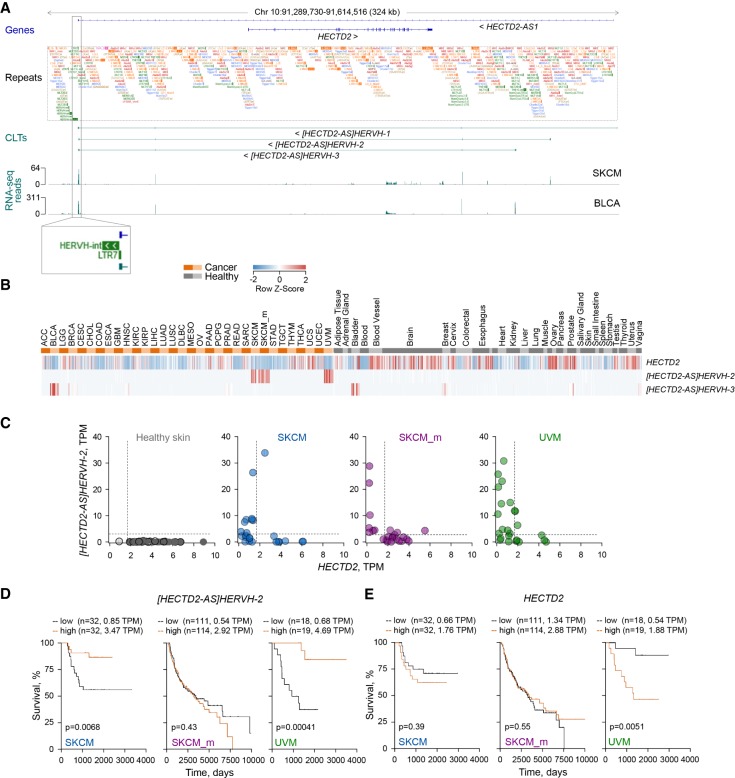
Down-regulation of *HECTD2* expression by melanoma-specific antisense transcription of the *[HECTD2-AS]HERVH-2* CLT. (*A*) GENCODE annotated transcripts at the indicated genomic location (genes), repeat content (repeats), CLTs and other selected transcripts at the same location in the current assembly (CLTs), and RNA-seq traces of representative SKCM and BCLA samples. (*B*) Heatmap of expression values in cancer patient and healthy control samples of *HECTD2* and the two indicated antisense transcripts. (*C*) Anticorrelation of *HECTD2* and *[HECTD2-AS]HERVH-2* expression (TPM values). Each symbol is an individual patient or healthy control sample. (*D*,*E*) Kaplan-Meier plots and *P*-values from log-rank tests for melanoma patients stratified according to the higher versus the lower expression tertiles for *[HECTD2-AS]HERVH-2* (*D*) and *HECTD2* (*E*). The number of cases and the expression thresholds are also indicated in brackets.

Context-dependent expression and association with survival probability was also observed for a novel transcript, [*HECTD2-AS*]*HERVH-2*, antisense to the *HECTD2* locus (Fig. 5A–E). This locus was found to produce a long antisense transcript [*HECTD2-AS*]*HERVH-1*, partially matching the annotated *HECTD2-AS1* lncRNA (NR_024467.1), but also two additional transcripts, [*HECTD2-AS*]*HERVH-2* and [*HECTD2-AS*]*HERVH-3*, through use of alternative TSSs (Fig. 5A), further supported by promoter-based expression analyses (Supplemental Fig. S5; The FANTOM Consortium and the RIKEN PMI and CLST (DGT) 2014). All three transcripts skipped exon 2 of the annotated *HECTD2-AS1* and terminated at a HERV-H LTR (Fig. 5A). [*HECTD2-AS*]*HERVH-1* was not significantly expressed in any sample, but [*HECTD2-AS*]*HERVH-2* was found highly expressed specifically in SKCM and UVM, and [*HECTD2-AS*]*HERVH-3* was expressed in BLCA, as well as healthy bladder and reproductive tissues (Fig. 5B). Melanoma-specific expression of [*HECTD2-AS*]*HERVH-2* was accompanied by loss of sense *HECTD2* transcription (Fig. 5C), and a similar loss of the protein-coding sense transcript effect was mediated by antisense transcription of [*HECTD2-AS*]*HERVH-3* in healthy bladder and BLCA (Supplemental Fig. S6), a pattern confirmed also in CCLE cell lines (Supplemental Fig. S7). The apparent switch from sense to antisense transcription was linked with better prognosis in primary SKCM and UVM, but not in metastatic SKCM, which showed higher expression of protein-coding *HECTD2* across all patients (Fig. 5D,E).

Contrasting prognostic association with primary SKCM or UVM and metastatic SKCM was observed also for the *MLT1B*[*LINC00518*]*-1* transcript (Supplemental Fig. S8), which matches a transcript previously described as the SKCM-specific lncRNA *LINC00518* (NR_027793.1) (Iyer et al. 2015) and which we found to be initiated by an MLT1B element in both SKCM and UVM (Supplemental Fig. S8). Better prognosis uniquely in primary SKCM was associated with higher expression of a standalone HERV-K integration, *HERVK3*[*Chr19q13.43*], despite its noticeable expression in many healthy tissues and further transcriptional induction in most cancer types (Supplemental Fig. S9). In comparison, survival probability specifically in metastatic SKCM positively correlated with higher expression of the well-studied *MER41G*[*AIM2*] transcript (Chuong et al. 2016) (Supplemental Fig. S10) and a novel transcript, [*DRAIC*]*LTR67B*, that partial overlaps with GENCODE transcript ENST00000559212 (*DRAIC-210*) (Supplemental Fig. S11), both showing an immune-related, rather than melanoma-specific, expression pattern.

Together, these results indicate diverse biological processes linking CLT expression clusters with disease progression, as well as the specificity of their transcriptional regulation in distinct cancer and tissue types, further increased in distinct disease subtypes.

### Unique cancer-specific antigens predictably generated by CLTs

**Figure 6.**
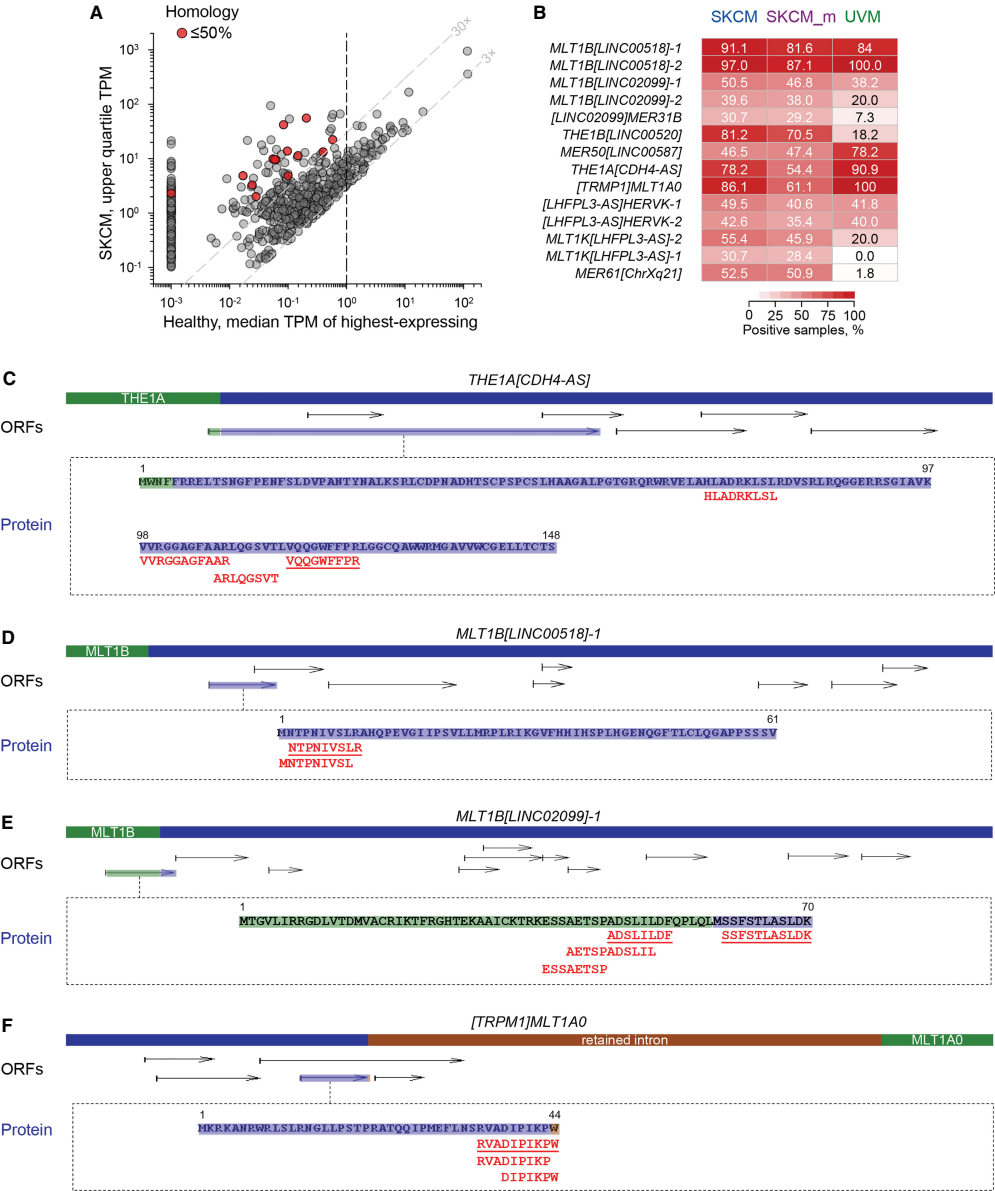
Potential antigenicity of SKCM-specific CLTs. (*A*) Properties of selected CLTs (red circles) with unique protein-coding potential. Plotted is the expression level of SKCM CLTs with a predicted ORF ≥300 nt, in SKCM (upper quartile TPM) against the median TPM of the highest-expressing healthy tissue. Although the cut-off for CLT selection was ≤85% homology over the entire ORF length with any other ORF potentially expressed in healthy tissues, the final selected CLTs displayed 0%–50% homology. (*B*) Prevalence of expression of the indicted CLTs with unique protein-coding potential among primary SKCM (*n* = 101), metastatic SKCM (SKCM_m; *n* = 342), or UVM (*n* = 55) patients. Values are the percentages of patient that express each CLT at 0.5 or more TPM. (*C*–*F*) CLT structure, all predicted ORFs >75 nt (ORFs), where the ORF with evidence for translation is highlighted, and amino acid sequence of the latter ORF. Also shown is the sequence of MHC-eluted peptides uniquely mapping to each CLT product. Underlined peptides were confirmed by comparison with synthetic peptides.

Regardless of the forces driving their expression in cancer, when expressed, CLTs may encode polypeptides that, irrespective of any intrinsic biological activity, are processed into unique antigenic peptides presented by MHC molecules. To identify CLTs with protein-coding potential, we used an ORF prediction algorithm, based on length and suitability of dicodon (hexamer) scores. We initiated our search in SKCM, ranking the transcripts according to expression and focusing on transcripts that contained one or more ORFs ≥300 nt. To ensure high cancer specificity, we eliminated CLTs with median levels of one or more TPM in the highest-expressing healthy tissue (Fig. 6A). Additionally, we removed transcripts whose largest ORF displayed >85% amino acid sequence identity with any other protein, annotated or predicted, that was potentially expressed in healthy tissues. This filter combination returned 14 CLTs, transcribed from eight different loci, each containing a unique ORF (0%–50% identity with other ORFs) (Fig. 6A,B). These SKCM-marking CLTs were expressed (one or more TPM) in between 46% and 99% of the extended SKCM cohort we have examined, and most of them were similarly expressed in UVM (Fig. 6B). The selected CLTs included the prognostic [*TRPM1*]*MLT1A0*, [*HECTD2-AS*]*HERVH-2*, and *MLT1B*[*LINC00518*]*-1* transcripts and additional transcripts with similar structure. Several transcripts highly expressed specifically in melanoma overlapped LTR elements in the *LHFPL3* locus, a gene whose expression was discordantly associated with survival of primary SKCM and UVM (Supplemental Fig. S12).

To probe for protein production by the selected SKCM-specific CLTs, we searched through immunopeptidomic data previously generated from melanoma biopsies (Bassani-Sternberg et al. 2016). Although these CLTs were selected for the presence of at least one sequence-unique ORF, they could in principle contain additional or alternative ORFs. We therefore included the translation of any ORF of a minimum 75 nt from these selected CLTs in the search, which was performed using the Mascot search engine (Perkins et al. 1999). This analysis identified a number of MHC-eluted peptides, with Mascot scores higher or close to the respective homology scores (Supplemental Table S6), that mapped to ORFs from CLTs transcribed from four of the selected loci (Fig. 6C–F). To confirm correct assignment of the observed peptides, spectra from at least one peptide per ORF were compared, by spectral angle analyses (Tabb et al. 2003), with spectra generated with synthetic peptides (Supplemental Fig. S13). To obtain further support, the same immunopeptidomic data were searched with a different search engine, PEAKS, assisted by de novo peptide sequencing (Zhang et al. 2012). This analysis identified nine of the 13 Mascot-identified peptides as highly significant, with an average false-discovery rate (FDR) of 1.5% (Supplemental Table S6), providing independent validation of the observed spectra (Supplemental Fig. S14). Moreover, several of the identified peptides were predicted to bind at least one major HLA allele, and in most cases, they were also predicted to bind to the HLA allele (where known) of the patient in whom they were identified (Supplemental Fig. S15). Collectively, these results supported the antigenicity of the CLT translation products.

Peptides were identified from the largest predicted ORF of transcript *THE1A*[*CDH4-AS*] (Fig. 6C). This transcript, matching the annotated lncRNA *RP11-429E11.2*, was found to be initiated by a *THE1A* element in intron 3 of the *CDH4* gene, driving transcription in the antisense orientation (Supplemental Fig. S16). The *THE1A*[*CDH4-AS*] transcript was very highly expressed specifically in SKCM and UVM but may also be expressed to a much lower degree in UV-exposed skin (Supplemental Fig. S16). Peptides were also identified from smaller ORFs of the prognostic transcript *MLT1B*[*LINC00518*]*-1* and transcript *MLT1B*[*LINC02099*]*-1* (Fig. 6D,E). The latter transcript was initiated by an *MLT1B* element and was one of three identified transcripts in the lncRNA *LINC02099* locus, matching the annotated *LINC02099-201* variant (Supplemental Fig. S17). Also identified were peptides derived from alternative translation of the prognostic [*TRPM1*]*MLT1A0* transcript. Although translation of the largest ORF in transcript [*TRPM1*]*MLT1A0* is supported by RefSeq evidence (annotated protein NP_001238959), eluted peptides were additionally derived from translation of a smaller embedded alternative ORF (Fig. 6F). Together, these results highlight the potential of CLT translation and presentation to contribute to the diversity of cancer-specific antigenic peptides.

## Discussion

By assembling a comprehensive transcriptome, we extend our knowledge of LTR elements that are transcriptionally used and their potential involvement in human cancer. Although accumulating evidence incriminates ERVs and other EREs in multiple stages of cancer development and anticancer immunity (Romanish et al. 2010; Kassiotis and Stoye 2017), their impact is not yet fully appreciated owing to the lack of complete annotation and quantitation of LTR element–overlapping transcripts. Past efforts involving mapping reads to the reference genome have reduced representation of repeat-derived exons owing to exclusion of multimapping reads and loci. Our assembly mitigates this bias because the majority of the genomic repeats are not expressed in a particular indication, increasing the likelihood of unambiguously assigning a repeat-derived read to an individual transcript during transcriptome-based assembly. This resulted in substantially increased representation of unannotated or partially annotated transcripts derived from or overlapping with LTR and LINE elements, in the current assembly. Although cancer-specific ERE-overlapping transcripts might not be biologically more significant than those that do not overlap with EREs, they do represent the overwhelming majority of novel transcripts in this assembly and were therefore the focus of this study, starting with LTR-overlapping transcripts.

Global epigenetic changes may be necessary for the transcriptional utilization of LTR elements in cancer. However, our data suggest that alone these changes are not sufficient. Instead, utilization of distinct LTR elements is locus specific and does not necessitate activation of other LTR elements of the same family. A number of observations support this notion. Regardless of cancer specificity, the expression of chimeric transcripts was significantly higher than that of stand-alone LTR elements. Furthermore, chimeric transcripts were overrepresented in the CLT pool, as exemplified in melanoma CLTs, in which they were one order of magnitude more numerous than stand-alone LTR elements. Indeed, derepression of LTR promoter activity could account for transcriptional induction of only a minority of CLTs, whereas transcriptional inclusion of LTR elements by alternative splicing in transcripts driven by alternative promoters was more frequent. These findings suggest a combination of LTR and gene properties is responsible for cancer-specific transcriptional induction of chimeric CLTs and may also underlie their tissue-type specificity. This is further supported by the close association of CLT expression and molecular disease subtypes, defined based on protein-coding gene expression alone (The Cancer Genome Atlas Network 2015; Robertson et al. 2017). This close link substantially increases the predictability of CLT expression in their respective disease subtype, often exceeding 90% of samples analyzed here. It also underlies previously unappreciated shifts in CLT expression mirroring cancer progression or metastasis. For example, the lower extent of overlap in CLT expression between individual samples within and between metastatic SKCM cohorts indicated a considerably higher degree of heterogeneity in metastatic than in primary SKCM. Such shifts in CLT expression likely reflect changes in overall gene expression, such as down-regulation of *TRPM1* and other MITF targets in metastatic SKCM (Duncan et al. 1998; The Cancer Genome Atlas Network 2015), or immune escape.

The combination of tissue and cancer specificity may also relate to cell lineage–specific transcription of CLTs. A confounding factor in determining cancer specificity of a given CLT by comparing its expression between tumor biopsies and healthy tissues is the relative proportion of tumor cell lineage in each sample. For example, a higher proportion of melanocytes in melanoma biopsies than in healthy skin might favor melanocyte-specific transcript selection. Indeed, expression of [*HECTD2-AS*]*HERVH-1* was detected in a nontransformed melanocyte cell line, as well as in melanoma cell lines, and expression of *THE1A*[*CDH4-AS*] was also found in sun-exposed healthy skin. Nevertheless, melanocytes are a sizable constituent of healthy skin, with frequencies between 10% and 50% dependent on anatomical location (Cochran 1970), and melanoma biopsies also contain a substantial fraction of nontransformed stromal and immune cells, which can add up to 50%. Furthermore, although detectable in a nontransformed melanocyte cell line and in sun-exposed healthy skin, respectively, expression of [*HECTD2-AS*]*HERVH-1* and *THE1A*[*CDH4-AS*] was still significantly elevated in melanoma cell lines and biopsies, supporting the notion that disregulated or exaggerated melanocyte-specific transcriptional patterns underlie melanoma specificity of at least some of the melanoma CLTs.

Although expression of most CLTs simply reflects the altered epigenetic, splicing, and transcriptional state of cancer, some will have functionally relevant implications. These include *cis* effects on gene transcriptional activity or splicing patterns, as represented by the *HECTD2* locus. First identified as a prion disease susceptibility gene (Lloyd et al. 2009), *HECTD2* encodes a ubiquitin E3 ligase that enhances the inflammatory response to innate stimulation (Coon et al. 2015). MicroRNA-mediated down-regulation of *HECTD2* expression has been proposed to drive androgen independence in prostate cancer (Sun et al. 2014), and more recently, *HECTD2* copy number alterations were suggested as drivers in neuroblastoma patients (Suo et al. 2018). However, the function of *HECTD2* in cancer in general and in melanoma in particular remains to be elucidated. Its transcriptional regulation by antisense CLT transcription in melanoma revealed here clearly illustrates the potential of our assembly of novel CLTs to identify biologically relevant genes, as well as their regulation, which will provide numerous leads for further investigation.

Another functional implication of CLT expression is the potential to generate cancer-specific antigens for T cell recognition. Cancer cells present antigenic T cell epitopes from canonical and noncanonical polypeptides, showing different degrees of frequency and specificity (Schumacher and Schreiber 2015). These range from highly immunogenic neoantigens, generated by cancer-specific mutations restricted to one or few individuals, to partially immunologically tolerated antigens from nonmutated canonical proteins overexpressed in almost all cancer patients. It is becoming increasingly clear that the altered transcriptional state of cancer can generate novel noncanonical polypeptides without the need for somatic mutations (Kahles et al. 2018; Smart et al. 2018). These include alternative splicing events, such as intron retention (Smart et al. 2018) or mid-exon splicing (Kahles et al. 2018) of protein-coding genes, and, as we show here, transcriptional utilization of LTR elements. Our analysis uncovered several transcripts overlapping LTR elements that are highly prevalent in distinct cancer types, some expressed in nearly all patients examined. This high predictability of CLT expression contrasts with that of transcripts generated by other mutation-independent (Kahles et al. 2018; Smart et al. 2018) or mutation-driven processes (Schumacher and Schreiber 2015) that occur at relatively low frequency. Thus, our findings uncover a novel source of cancer antigens, with characteristics of expression specificity and intensity that predict a distinctive immunological profile. Although their antigenicity is shown here, further studies will be needed to establish their immunogenicity or therapeutic potential.

Despite their mutation-independent nature, CLTs have the potential to produce completely novel antigenic peptides, translated from LTR-initiated transcripts previously considered to lack coding potential (e.g., lncRNAs), chimeric ORFs resulting from LTR element exonization or alternative ORFs in LTR-overlapping transcripts, as well as ORFs in stand-alone LTR elements. Although protein products from these CLTs have not been previously observed, recent in-depth proteogenomic analysis of an Epstein-Barr virus–transformed B cell line (Laumont et al. 2016) or A431 cells and five healthy tissues (Zhu et al. 2018) provided evidence for the translation of similarly unconventional ORFs, including lncRNAs, alternative ORFs, and intronic sequences. Translation of pseudogenes and lncRNAs also showed tissue specificity (Zhu et al. 2018). Moreover, a recent analysis of the murine cancer immunopeptidome highlighted noncoding regions as the main source of cancer-specific antigens (Laumont et al. 2018). As our analysis of the melanoma immunopeptidome shows, such unconventional transcripts and ORFs therein hold great promise for cancer-specific antigens.

Although the annotated LTR transcriptome has been increasingly implicated in cancer (Romanish et al. 2010; Kassiotis and Stoye 2017), the broader view of LTR element transcriptional utilization offered by the current assembly reveals a much more extensive involvement of these elements than previously appreciated. We expect that the identification and quantitation of the cancer LTR transcriptome in this study will provide a framework for fully investigating and elucidating their role in cancer initiation, progression, and immune surveillance.

## Methods

### Repeat region annotation

LTR and non-LTR elements were annotated as described previously (Attig et al. 2017) and in the Supplemental Methods.

### Transcript assembly

RNA-seq reads from 24 patient samples from 31 primary and one metastatic (melanoma) cancer types (totaling 768 samples) were obtained from TCGA (Supplemental Table S1) and used to generate a pan-cancer transcriptome, as described in full in the Supplemental Methods (see also Supplemental File S1; Supplemental Code S3).

### Cancer-specific transcript selection

TPMs were estimated for all transcripts with a custom Bash pipeline (Supplemental Code S1) using GNU parallel (Tange 2011) and Salmon, and expression within each cancer type was compared with expression across 811 healthy tissue samples (healthy tissue-matched controls for all cancer types, where available, from TCGA and the GTEx Consortium) (The GTEx Consortium 2015). RNA-seq data from both these consortia are based on poly(A) RNA, which, as opposed to total RNA, may affect the capture of certain classes of transcripts. However, poly(A) RNA selection was recently shown to limit detection only of satellite repeat transcripts, whereas transcripts from ERVs, LINEs, and SINEs were similarly represented in total and poly(A)-selected RNA (Solovyov et al. 2018). Moreover, the 3′ UTR and thus the poly(A) tail in the majority of CLTs are provided by the canonical fusion gene rather than the LTR element. Therefore, the impact of using poly(A)-selected RNA-seq samples on our ability to detect and quantify LTR-overlapping transcripts should be negligible.

Transcripts were considered expressed if detected at more than one TPM in any sample and as cancer-specific if the following criteria were fulfilled: (1) expressed in six or more of the 24 samples of each cancer type; (2) expressed at less than 10 TPM in ≥90% of all healthy tissue samples; (3) expressed in the cancer type of interest three or more times the median expression in any control tissue type; and (4) expressed in the cancer type of interest three or more times the 90th percentile of the respective healthy tissue, when available (Supplemental Code S2). In addition to these expression thresholds, transcript selection was based on manual inspection, excluding potentially misassembled contigs. When the DNA strand could not be unambiguously assigned (using a range of criteria, including overlap with annotated exons, directionality of LTRs, splice or polyadenylation sites), transcripts corresponding to both strands were considered.

### Survival analysis and hazard ratio calculations

For survival analysis, we downloaded the complete cohorts of SKCM and UVM from TCGA, for which survival data were recorded (primary SKCM: 95; metastatic SKCM: 335; UVM: 54). To test if expression of a transcript of interest correlated with patient survival, we identified the patients in the bottom and top tercentile expression (“low” vs. “high” expression). Survival analysis was performed using the *survfit* function of the survival R package (v. 2.42) (R Core Team 2018), using overall survival time based on the days_to_death annotation or the last update of the annotation in March 2017 (i.e., 365 days × (2017 − year of birth) − the age-at-diagnosis in days). To compare curves between low and high expression tertiles, log-rank testing was used, and a Cox regression model was built to test the assumption of proportional hazards holds. Hazard odd ratios are given based on the Cox regression model. To compare survival between multiple expression clusters, the Cox regression model was used.

### Unique protein-coding potential prediction

To identify CLTs with protein-coding potential, we ran an ORF prediction algorithm, based on length and suitability of dicodon (hexamer) scores. A HMM was trained on hexamers derived from Ensembl CDS annotations, and ORFs ≥300 nt were taken forward, where their sense hexamer score exceeded the antisense score. To identify unique protein sequences potentially encoded by CLTs, sequences translated from the largest ORF of selected CLTs were aligned with those translated from all ORFs ≥210 nt from the entire transcript assembly using TBLASTN (BLAST+ v2.3.0) without soft-masking and retaining alignments with an *E*-value >10^−5^. Uniqueness was defined as ≤85% amino acid sequence identity (over the entire length of the protein) with any other predicted protein, whether the latter was expressed or not. For CLT-encoded proteins that showed >85% sequence identity with one or more predicted proteins, we interrogated the expression pattern of transcripts encoding the similar proteins. Where these additional transcripts were also expressed in a cancer-specific manner (based on the criteria listed above), the respective CLT was retained. Where the additional transcripts were expressed in healthy tissue, the respective CLT was discarded.

### Immunopeptidomic analyses

CLT-encoded HLA-presented peptides were identified by immunopeptidomic analyses of previously generated spectrometry data (accession number: PXD004894) (Bassani-Sternberg et al. 2016), as detailed in the Supplemental Methods (see also Supplemental File S2).

## Competing interest statement

G.K. is a scientific cofounder of and consulting for ERVAXX and a member of its scientific advisory board. G.R.Y. consults for ERVAXX. N.T. is currently directing immunopeptidomics research at ERVAXX. Part of this work was included in a patent application (ERV-P2166EPp).

## Supplementary Material

Supplemental Material
