# Supplementary material for: LTR retroelement expansion of the human cancer transcriptome and immunopeptidome revealed by de novo transcript assembly
Source: Genome Res. 2019 Oct;29(10):1578–90. doi: 10.1101/gr.248922.119 (PMC6771403; doi:10.1101/gr.248922.119)
Supplement: Supplemental Material [file supp_29_10_1578__index.html]

LTR retroelement expansion of the human cancer transcriptome and immunopeptidome revealed by de novo transcript assembly — LTR retroelement expansion of the human cancer transcriptome and immunopeptidome revealed by de novo transcript assembly — Supplemental Material 

# LTR retroelement expansion of the human cancer transcriptome and immunopeptidome revealed by de novo transcript assembly

## Supplemental Material

- Supplemantal\_Methods.pdf
- Supplemental\_Figures.pdf
- Supplemental\_Tables.xlsx
- Supplemental\_File\_S1.tar
- Supplemental\_File\_S2.txt
- Supplemental\_Code\_S1.docx
- Supplemental\_Code\_S2.R
- Supplemental\_Code\_S3.R
