# Supplementary material for: LTR retroelement expansion of the human cancer transcriptome and immunopeptidome revealed by de novo transcript assembly
Source: Genome Res. 2019 Oct;29(10):1578–90. doi: 10.1101/gr.248922.119 (PMC6771403; doi:10.1101/gr.248922.119)
Supplement: Supplemental Material [file supp_gr.248922.119_Supplemantal_Methods.pdf]

Supplemental Methods

Repeat region annotation

LTR and non-LTR elements were annotated as previously described (Attig et al. 2017). Briefly, hidden Markov models (HMMs) representing known Human repeat families (Dfam 2.0 library v150923) were used to annotate GRCh38 using RepeatMasker (Smit et al. 2013-2015), configured with nhmmer (Wheeler and Eddy 2013). HMM-based scanning increases the accuracy of annotation in comparison with BLAST-based methods (Hubley et al. 2016). RepeatMasker annotates LTR and internal regions separately, thus tabular outputs were parsed to merge adjacent annotations for the same element.

Transcript assembly

RNA-seq reads from 24 patient samples from 31 primary and 1 metastatic (melanoma) cancer types (totalling 768 samples) were obtained from TCGA (Supplemental_Table_S1) and used to generate a pan-cancer transcriptome. The data were obtained from dbGaP accession numbers phs000178.v10.p8.c1 and phs000424.v7.p2.c1 in 2017. For individual cancer types, data from 24 gender-balanced samples (excluding gender-specific tissues) were adapter and quality (Q20) trimmed and length filtered (both reads of the pair ≥35 nucleotides) using cutadapt (Marcel 2011) (v1.13) and kmer-normalized (k=20) using khmer (Crusoe et al. 2015) (v2.0) for maximum and minimum depths of 200 and 3, respectively. Reads were mapped to GRCh38 using STAR (2.5.2b) with settings identical to those used across TCGA and passed to Trinity (Grabherr et al. 2011) (v2.2.0) for a genome-guided assembly with inbuilt *in silico* depth normalization disabled. The majority of assembly processes completing within 256GB RAM on 32-core HPC nodes, with failed processes re-run using 1.5TB RAM nodes. Resulting contigs were poly(A)-trimmed (trimpoly within SeqClean v110222) and entropy-filtered (≥0.7) to remove low-quality and artefactual contigs (bbduk within BBMap v36.2). Per cancer type, the original 24 samples were quasi-mapped to the cleaned assembly using Salmon (v0.8.2 or v0.9.2), with contigs found expressed at <0.05 transcripts per million (TPM) being removed. Those remaining were mapped to GRCh38 using GMAP (Wu and Watanabe 2005) (v161107), filtering out contigs not aligning with ≥85% identity over ≥85% of their length. Finally, assemblies for all cancer types together were flattened and merged into the longest continuous transcripts using gffread (Cufflinks v2.2.1) (Trapnell et al. 2010). As this assembly process was

specifically designed to enable assessment of repetitive elements, monoexonic transcripts were retained, but flagged.

The transcript assembly was annotated against GENCODE (basic, version 24) (Frankish et al. 2018), using cuffcompare at default settings (Cufflinks v2.2.1) (Trapnell et al. 2010) and a custom script (Supplemental_Code_S3), using R (R Core Team 2018). Any exon that was perfectly identical between the two annotations was assigned the GENCODE exon ID and flagged. Where possible, we assigned transcripts a strand based on the overlap with GENCODE annotation, in that every transcript with one or more exons identical to GENCODE exons was assigned the strand of those exons. For exons that overlapped a GENCODE exon partially, we recorded annotation from GENCODE (gene IDs, gene level, gene and transcript type, Ensembl transcript IDs) if (i) the transcript assembly exon was entirely contained within a GENCODE exon or (ii) at least 10% of the exon overlapped with a GENCODE exon, prioritizing the GENCODE exon(s) with the largest overlap. Based on the overlap with GENCODE exons, we assigned features to each transcript from GENCODE, such as Ensembl transcript IDs, the gene and transcript type (protein-coding, lncRNA, etc.), the gene symbol (according to HUGO Gene Nomenclature Committee), the transcript support level in GENCODE, and the number of exons identical or partially overlapping GENCODE exons. To assign gene and transcript features to transcripts with exons that overlapped multiple GENCODE exons of different origin, we prioritized GENCODE transcripts by the transcript support level and assigned protein-coding entries, lncRNAs, and other features hierarchically, in this order. In addition, we recorded features from the transcript assembly including total exon number, open reading frames and overlap with any RepeatMasker repeat. From the custom R script (Supplemental_Code_S3), we regenerated an annotated GTF file for downstream analysis (Supplemental_File_S1).

Transcript assembly completeness and quality was assessed by comparison with GENCODE (Frankish et al. 2018) and MiTranscriptome (Iyer et al. 2015). We compiled the list of unique splice sites represented within GENCODE and tested if the splice site was present within the transcriptome assembly within a 2-nucleotide grace window.

Immunopeptidomic analyses

Raw mass spectrometry data previously generated from immunopeptidomic analysis of melanoma biopsies (Bassani-Sternberg et al. 2016) (Accession number: PXD004894), were retrieved from the Proteomics Identifications (PRIDE) database (https://www.ebi.ac.uk/pride) for further interrogation.

As protein products from the selected CLTs were not previously annotated, these were appended to the search database. The translation of any ORF ≥75 nucleotides (including the stop codon) present

in 23 selected melanoma-specific CLTs, resulting in 205 additional sequences ranging from 23 to 509 amino acids, were used in the analyses (Supplemental_File_S2). Where transcript strand was known, only ORFs present on the correct strand were included, whereas where transcript strand was not known, ORFs present on either strand were considered.

The data were searched using the Mascot search engine (v2.3.1) and a database of the translation of CLT ORFs (Supplemental_File_S2) appended to the known human proteome, using the FASTA file which was also used in the first description of the immunopeptidomic data (Bassani-Sternberg et al. 2016). The following settings were used: Fixed modifications: none; Variable modifications: Oxidation (M), Acetyl (N-term), Phospho (STY); Missed cleavages: 2; MSMS tolerance: 0.5 Da; MS tolerance: 2 Da; and Enzyme specificity: set to non-specific. The top-scoring peptide spectrum matches (PSMs) per spectrum (up to 10, if more than 10 matches were identified) were saved and are shown in Supplemental_Table_S7.

Separately, data were searched with PEAKS v8.5 (Bioinformatics Solutions). Raw files from PXD004894 were imported into PEAKS and *de novo*-assisted spectral interpretation was performed using a database consisting of the reviewed SWISS-PROT human proteome (download on 5/12/2017, 20,255 entries) in concatenation with the translation of CLT ORFs (Supplemental_File_S2). Precursor mass tolerance was set to 10-20 ppm, according to the mass deviation of the instrument at time of measurement, fragment mass tolerance was set to 0.05 Da. No enzyme specificity was selected. At the chosen score threshold of 15, the false discovery rate was estimated to be an average of 1.5% (1.0%-1.9%) on peptide spectrum match level, as determined by simultaneous decoy database searches using the decoy-fusion approach that is integrated in the Peaks v8.5 software. PEAKS-identified peptides are listed in full in Supplemental_Table_S8.

Uniqueness of the identified peptides to the assigned ORF was tested using BLASTP (BLAST+ v2.3.0), without soft masking, against a protein database built from the transcriptome assembly including all ORFs above 70aa present on the correct strand of the transcript (where strand was known) or either strand (where strand was not known), and the Matrix Science Mascot Sequence database (MSDB) (http://proteomics.bio21.unimelb.edu.au/msdb), a composite database of non-identical entries, built from a number of source databases (UniProt, GenBank translations, RefSeq and PDB). BLASTP settings were optimized for short peptides by ensuring BLASTP finds for each peptide the ORF within the transcriptome assembly it was identified from. We found the PAM30 matrix with the following settings optimal for the purpose: -evalue 100 -word_size 2 -gapopen 9 -gapextend 1 -threshold 11. Peptides that uniquely mapped to their assigned ORF were kept.

HLA I-associated peptides shorter than 7 amino acids were ignored, whereas 7-mers were kept only as supporting sequence evidence for longer versions of the sequence, provided that latter were also detected. For example, the single 7-mer peptide we identified (DIPIKPW) was embedded in the 10-mer RVADIPIKPW peptide, which was also identified in two patients. Shorter peptides may represent degradation products of a longer epitopes and might not necessarily bind HLA. However, although rarer and possibly of lower binding affinity, functional 7-mer peptide epitopes have been described in several viral proteins, such as Influenza Virus matrix, Hepatitis B Virus core Ag and Human Papillomavirus E6 (Li et al. 2005; Nakagawa et al. 2007; Wahl et al. 2009) or in cancer, such as mucin 1 in human breast cancer (Apostolopoulos et al. 1997).

Correct spectra assignment was confirmed by comparing spectra of selected identified peptides with those generated with synthetic peptides. Peptides were synthesized on an Intavis Multipep Peptide Synthesiser (Intavis Bioanalytical Instruments AG, Cologne, Germany) on Wang resins, using standard FMOC chemistry and HCTU for coupling. Following cleavage and deprotection, then lyophilization, peptides were purified on a Perkin Elmer Series 200 system using a C8 reverse phase column and analyzed for mass and purity using an Agilent 1100 LC-MS system. For mass spectrometry analysis, synthetic peptides were re-suspended in 0.1 % TFA and loaded onto 50-cm Easy Spray column (Thermo Fisher Scientific). Reverse phase chromatography was performed using the RSLC nano U3000 (Thermo Fisher Scientific) with a binary buffer system at a flow rate of 250 nl/min. The solubilized peptides were run on a linear gradient of solvent B (2-30 %) in 34 minutes, total run time of 60 minutes including column conditioning. The nanoLC was coupled to a Q Exactive mass spectrometer using an EasySpray nano source (Thermo Fisher Scientific). The Q Exactive was operated in data-dependent mode acquiring HCD MS/MS scans (R=17,500) after an MS1 scan (R=70, 000) on the 3 most abundant ions using MS1 target of 1×10^6 ions, and MS2 target of 2×10^5 ions. The maximum ion injection time utilized for MS2 scans was 300 ms, the dynamic exclusion was set at 10 s, and the peptide match and isotope exclusion functions were enabled. To achieve optimal fragmentation a range of HCD collision energies were tested (25, 27, 28 and 30). Spectra corresponding to the synthetic peptides were scored with Mascot and further manual verification was performed using mirror plots. Correct match of observed and synthetic peptide spectra was additionally confirmed by spectral angle calculations, as previously described (Tabb et al. 2003), using a value of 60 as the significance threshold for spectral similarity.

The filters we used in our immunopeptidomic analyses identified 13 HLA I-associated epitopes (Supplemental_Table_S6), derived from ORFs in 4 transcripts that were specifically and recurrently expressed in melanoma patients. These were selected owing to their equivalence (in terms of immunological targetability) to neoantigens. Whilst focusing on the melanoma targetable

immunopeptidome, our analysis was not restricted to that. We considered these 13 epitopes as evidence that the 4 ORFs, from which they derived were expressed and translated. The number of distinct epitopes from the translation products of these 4 ORFs was limited by the HLA diversity of the patient samples. As different HLA alleles may present different peptide epitopes from these 4 ORFs, the number of epitopes will increase with the diversity of HLA molecules that can present them. Moreover, these 4 ORFs were selected (based on targetability criteria) from a much larger pool of transcripts (5,923), expressed specifically in one or more cancer types, and an even larger pool of transcripts (130,389) expressed in cancer, but also in some healthy tissues. If 4 ORFs can result in 13 peptide epitopes in melanoma patients (with limited HLA diversity), then 130,389 ORFs will result in many more peptide epitopes, even if these are not necessarily useful in anti-cancer immunity.

Algorithms used for the prediction of CLT peptides and their affinity for HLA allotypes

The affinity of the eluted CLT peptides for HLA allotypes was predicted using the NetMHC4.0 and NetMHCpan4.0 methods (http://www.cbs.dtu.dk/services) (Andreatta and Nielsen 2016). Allotypes were restricted to the HLA-A/-B supertype and common HLA-C alleles provided in the software (Andreatta and Nielsen 2016). The threshold for binding was set to 2% to include weak binders, with strong binders considered as those with a binding rank of <0.5% (standard settings in NetMHC4.0 and NetMHCpan4.0). Where no predicted binder was found within the HLA supertype, the CLT peptide sequence was used as input into the 'Immune Epitope Database Analysis Resource', which utilizes a broader HLA reference set (Weiskopf et al. 2013). The respective HLA allotype was then appended to the NetMHC4.0 database and peptide binding validated. Our analysis also identified HLA I-associated peptides ending with Proline. The original analysis of the PXD004894 immunopeptidomic data (Bassani-Sternberg et al. 2016) identified over 400 epitopes (counting 8-10-mers only) ending in Proline and our re-analysis was entirely consistent with this. Moreover, inspection of the peptides deposited at IEDB (www.iedb.org) showed that of 154,815 sequences present (counting 9-mers only), 1,395 end with Proline. Of these, NetMHCpan 4.0 analysis predicted 319 to bind mainly to HLA-B supertypes (B07 and B40). Therefore, we found no evidence to suggest that ending in Proline is not compatible with HLA binding. To verify that eluted peptides corresponded to the predicted processed peptides from the ORF candidate transcript sequences, the total ORF sequences were used as input for NetMHC4.0. All CLT ORF candidates yielded the eluted peptides as predicted peptides.

Custom Scripts

We made use of the following custom programs deposited at github.com/A-N-Other/pedestal: deinterleave (commit 169dd81), interleavei (commit 800e78f), and orf_scanner (commit f96ed8c). All programs are available under the permissive MIT license and we encourage code re-use and comment.

Supplemental Methods References

Andreatta M, Nielsen M. 2016. Gapped sequence alignment using artificial neural networks: application to the MHC class I system. *Bioinformatics* **32**: 511-517.

Apostolopoulos V, Karanikas V, Haurum JS, McKenzie IF. 1997. Induction of HLA-A2-restricted CTLs to the mucin 1 human breast cancer antigen. *Journal of immunology (Baltimore, Md : 1950)* **159**: 5211-5218.

Attig J, Young GR, Stoye JP, Kassiotis G. 2017. Physiological and Pathological Transcriptional Activation of Endogenous Retroelements Assessed by RNA-Sequencing of B Lymphocytes. *Front Microbiol* **8**: 2489.

Bassani-Sternberg M, Braunlein E, Klar R, Engleitner T, Sinitcyn P, Audehm S, Straub M, Weber J, Slotta-Huspenina J, Specht K et al. 2016. Direct identification of clinically relevant neoepitopes presented on native human melanoma tissue by mass spectrometry. *Nat Commun* **7**: 13404.

Crusoe MR, Alameldin HF, Awad S, Boucher E, Caldwell A, Cartwright R, Charbonneau A, Constantinides B, Edvenson G, Fay S et al. 2015. The khmer software package: enabling efficient nucleotide sequence analysis. *F1000Res* **4**: 900.

Frankish A, Diekhans M, Ferreira AM, Johnson R, Jungreis I, Loveland J, Mudge JM, Sisu C, Wright J, Armstrong J et al. 2018. GENCODE reference annotation for the human and mouse genomes. *Nucleic Acids Res* doi:10.1093/nar/gky955.

Grabherr MG, Haas BJ, Yassour M, Levin JZ, Thompson DA, Amit I, Adiconis X, Fan L, Raychowdhury R, Zeng Q et al. 2011. Full-length transcriptome assembly from RNA-Seq data without a reference genome. *Nat Biotechnol* **29**: 644-652.

Hubley R, Finn RD, Clements J, Eddy SR, Jones TA, Bao W, Smit AF, Wheeler TJ. 2016. The Dfam database of repetitive DNA families. *Nucleic Acids Res* **44**: D81-89.

Iyer MK, Niknafs YS, Malik R, Singhal U, Sahu A, Hosono Y, Barrette TR, Prensner JR, Evans JR, Zhao S et al. 2015. The landscape of long noncoding RNAs in the human transcriptome. *Nat Genet* **47**: 199-208.

Li H, Zhou M, Han J, Zhu X, Dong T, Gao GF, Tien P. 2005. Generation of murine CTL by a hepatitis B virus-specific peptide and evaluation of the adjuvant effect of heat shock protein glycoprotein 96 and its terminal fragments. *Journal of immunology (Baltimore, Md : 1950)* **174**: 195-204.

Marcel M. 2011 Cutadapt removes adapter sequences from high-throughput sequencing reads. *EMBnetjournal* **17**: 3.

Nakagawa M, Kim KH, Gillam TM, Moscicki AB. 2007. HLA class I binding promiscuity of the CD8 T-cell epitopes of human papillomavirus type 16 E6 protein. *Journal of virology* **81**: 1412-1423.

Smit AFA, Hubley R, Green P. 2013-2015. RepeatMasker Open-4.0. www.repeatmasker.org.

R Core Team. 2018. R: A Language and Environment for Statistical Computing. *R Foundation for Statistical Computing*: www.R-project.org.

Tabb DL, MacCoss MJ, Wu CC, Anderson SD, Yates JR, 3rd. 2003. Similarity among tandem mass spectra from proteomic experiments: detection, significance, and utility. *Analytical chemistry* **75**: 2470-2477.

Trapnell C, Williams BA, Pertea G, Mortazavi A, Kwan G, van Baren MJ, Salzberg SL, Wold BJ, Pachter L. 2010. Transcript assembly and quantification by RNA-Seq reveals unannotated transcripts and isoform switching during cell differentiation. *Nat Biotechnol* **28**: 511-515.

Wahl A, Schafer F, Bardet W, Buchli R, Air GM, Hildebrand WH. 2009. HLA class I molecules consistently present internal influenza epitopes. *Proceedings of the National Academy of Sciences of the United States of America* **106**: 540-545.

Weiskopf D, Angelo MA, de Azeredo EL, Sidney J, Greenbaum JA, Fernando AN, Broadwater A, Kolla RV, De Silva AD, de Silva AM et al. 2013. Comprehensive analysis of dengue virus-specific

responses supports an HLA-linked protective role for CD8+ T cells. *Proc Natl Acad Sci USA* **110**: E2046-2053.
Wheeler TJ, Eddy SR. 2013. nhmmer: DNA homology search with profile HMMs. *Bioinformatics* **29**: 2487-2489.
Wu TD, Watanabe CK. 2005. GMAP: a genomic mapping and alignment program for mRNA and EST sequences. *Bioinformatics* **21**: 1859-1875.
