# Supplementary material for: LTR retroelement expansion of the human cancer transcriptome and immunopeptidome revealed by de novo transcript assembly
Source: Genome Res. 2019 Oct;29(10):1578–90. doi: 10.1101/gr.248922.119 (PMC6771403; doi:10.1101/gr.248922.119)
Supplement: Supplemental Material [file supp_gr.248922.119_Supplemental_Figures.pdf]

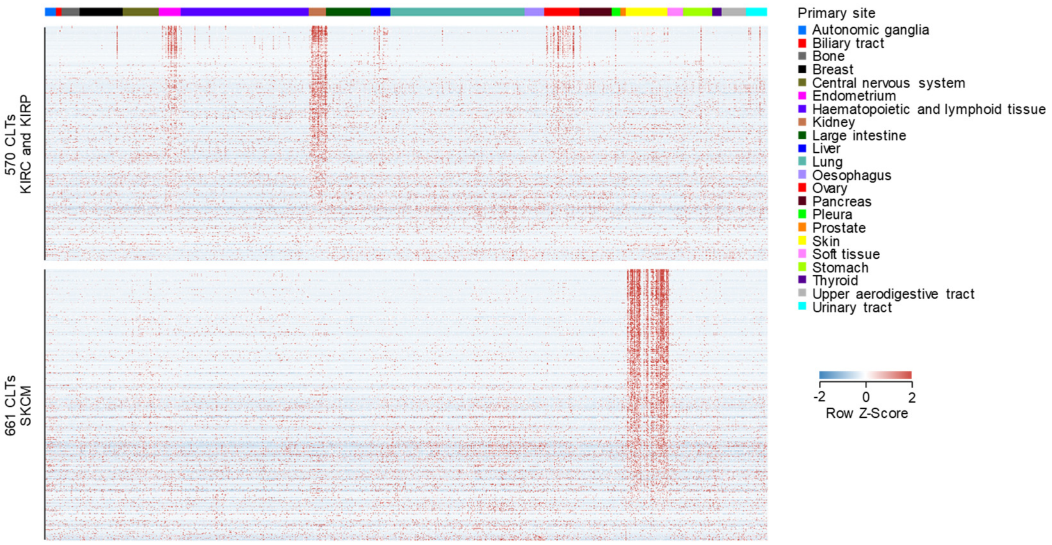

Supplemental Fig S1 Cancer histotype specificity of CLTs expression in cancer cell lines. Heatmap of expression values of 570 CLTs specific to KIRC or KIRP (*top*) and 661 CLTs specific to SKCM (*bottom*) in 935 CCLE cell lines of the indicated histotype.

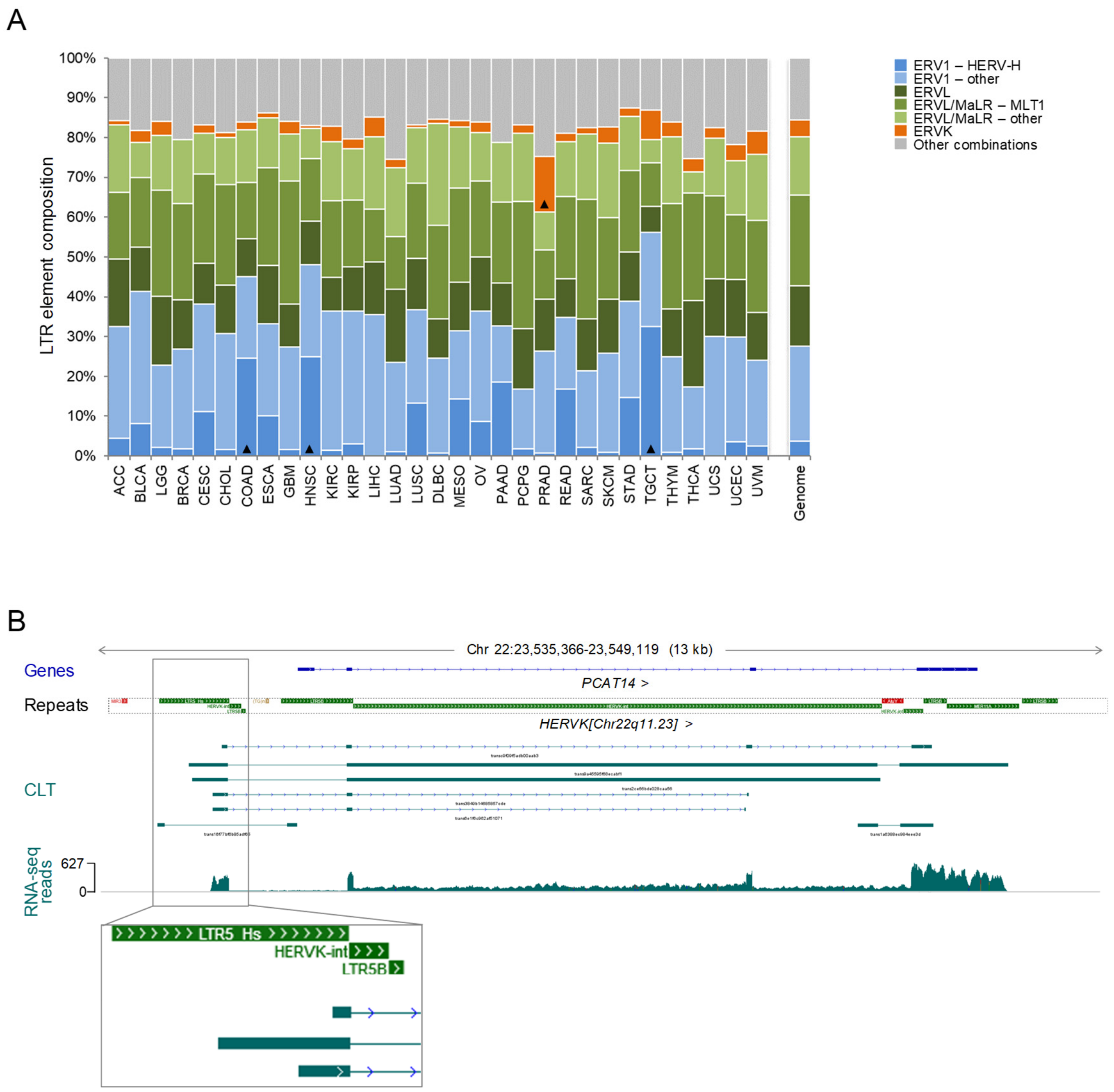

Supplemental Fig S2 LTR element composition of CLTs. (**A**) Stack bars indicate the percentage of LTR element families of CLTs expressed in the indicated cancer type, in comparison with their representation in the genome. Black triangles indicate over-represented families. (**B**) GENCODE annotated transcripts at the *PCAT14* genomic location (Genes), repeat content (Repeats), indicating the genomic structure of provirus *HERVK[Chr22q11.23]*, structure of several transcripts spanning that locus in the current assembly (CLT) and RNA-seq trace of a representative PRAD sample.

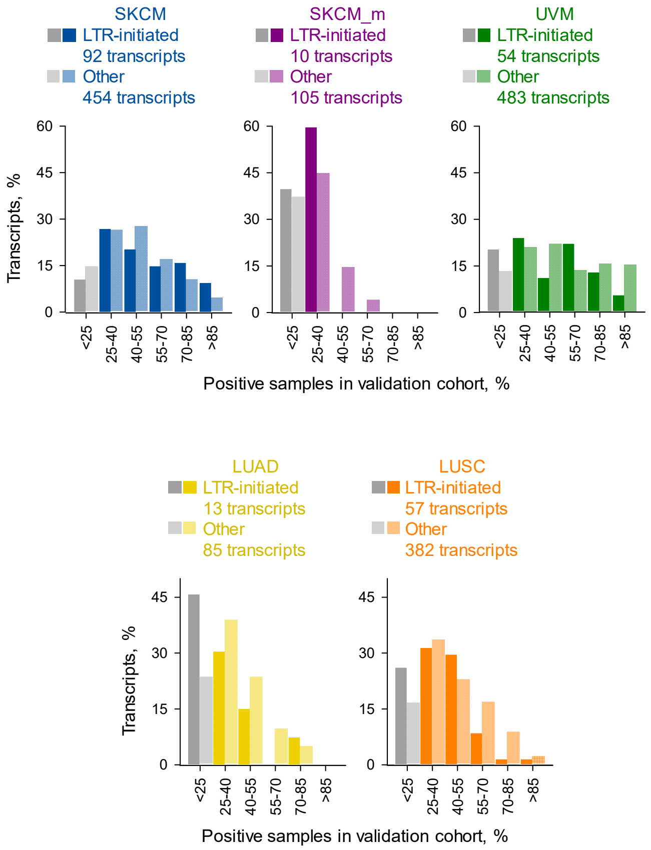

Supplemental Fig S3 Validation of CLT expression prevalence according to LTR position. Percentage of LTR-initiated and remaining CLTs, expressed in each bin of percentage of positive samples in cohorts of primary SKCM (n=77), metastatic SKCM (SKCM_m; n=318), UVM (n=31), LUAD (n=395) or LUSC (n=338). Samples were considered positive if transcript expression level was more than three times that of the highest median in any normal tissue.

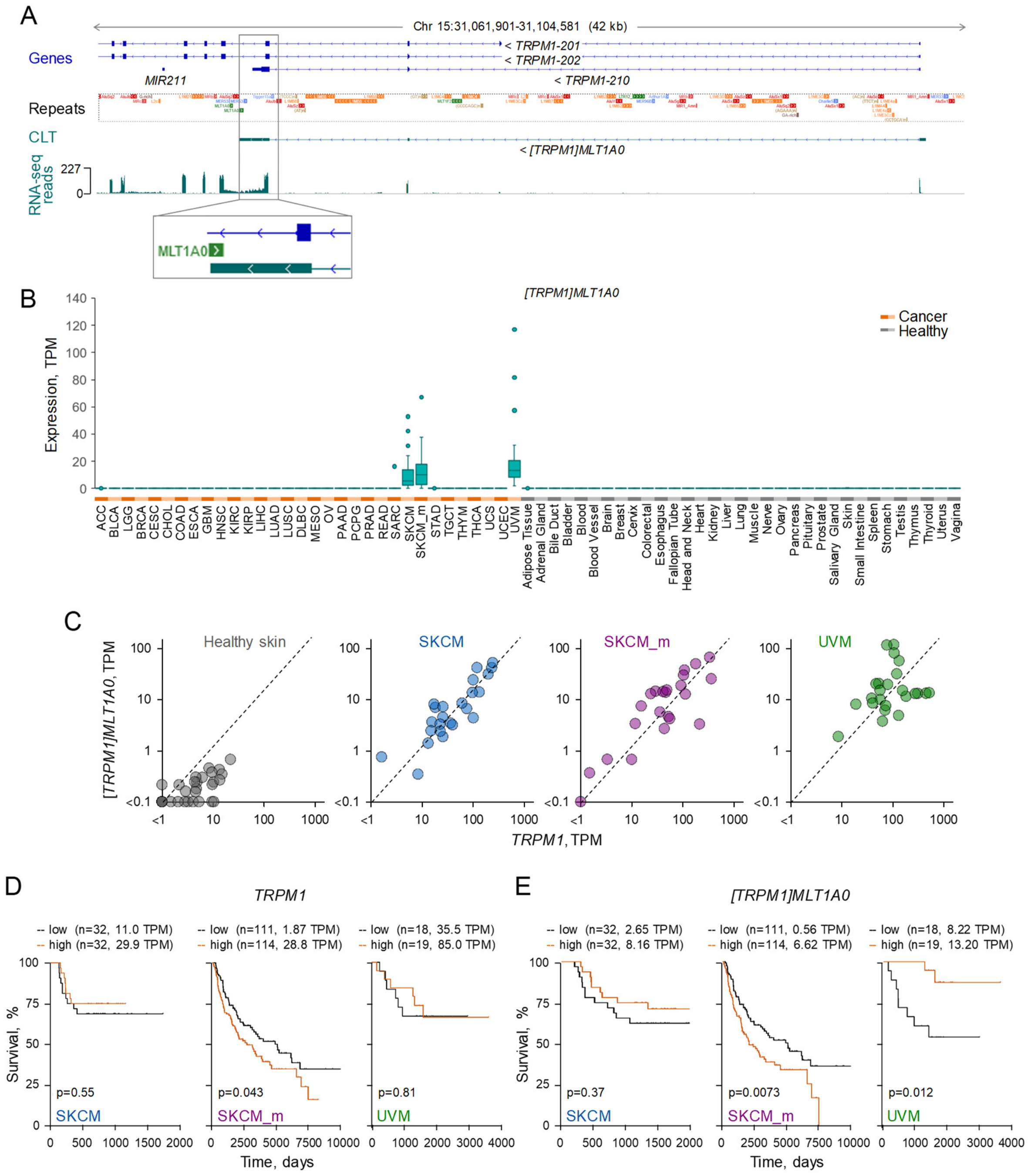

Supplemental Fig S4 Truncated *TRPM1* transcript created by alternative splicing and MaLR element exonisation. (**A**) GENCODE annotated transcripts at the indicated genomic location (Genes), repeat content (Repeats), structure of the *[TRPM1]MLT1A0* transcript in the current assembly (CLT) and RNA-seq trace of a representative SKCM sample. (**B**) Box plot of *[TRPM1]MLT1A0* expression in cancer patient and healthy control samples. (**C**) Correlation of *TRPM1* and *[TRPM1]MLT1A0* expression (TPM values). Each symbol is an individual patient or healthy control sample. (**D**, **E**) Kaplan-Meier plots and p-values from log-rank tests for melanoma patients stratified according to the higher versus the lower expression tertiles for *TRPM1* (D) and *[TRPM1]MLT1A0* (E). The number of cases and the expression thresholds are also indicated in brackets.

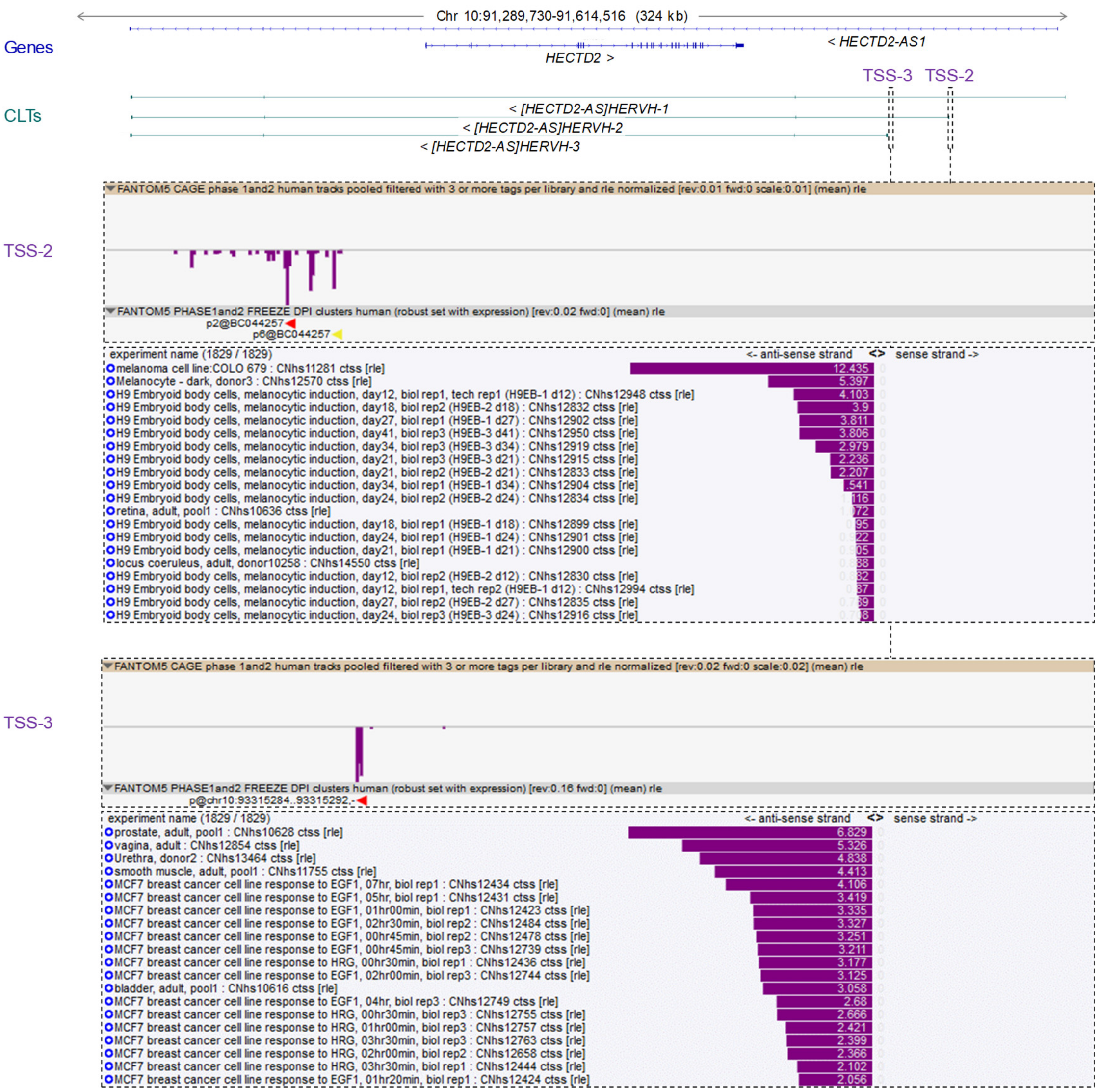

Supplemental Fig S5 CAGE support for transcription start sites of antisense transcripts at the *HECTD2* locus. Normalized data from the FANTOM Consortium and the RIKEN PMI and CLST (DGT) for transcription start sites 2 (TSS-2) and 3 (TSS-3) (both depicted in 1.4 Kb intervals), initiating transcripts *[HECTD2-AS]HERVH-2* and *[HECTD2-AS]HERVH-3*, respectively.

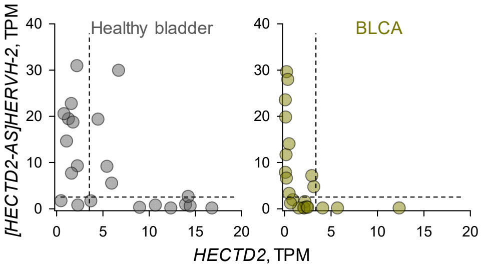

Supplemental Fig S6 Anti-correlation *HECTD2* and *[HECTD2-AS]HERVH-2* expression BLCA and normal bladder samples. Plotted are the TPM values for each sample. Each symbol is an individual patient or healthy control sample.

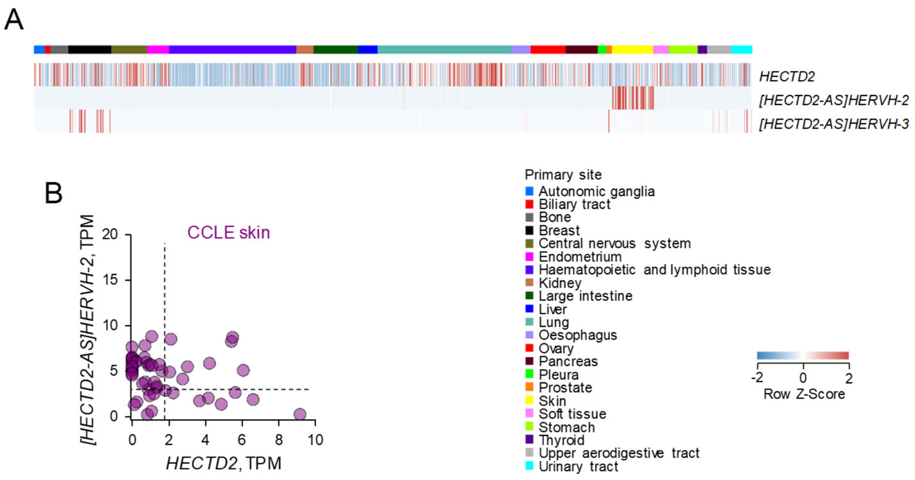

Supplemental Fig S7 Expression of *HECTD2* and of the antisense transcripts in cancer cell lines. (**A**) Heatmap of expression values of *HECTD2*, *[HECTD2-AS]HERVH-2* and *[HECTD2-AS]HERVH-3*, in 935 CCLE cell lines of the indicated histotype. (**B**) Anti-correlation *HECTD2* and *[HECTD2-AS]HERVH-2* expression (TPM values) in the same cells lines. Each symbol is an individual cell line.

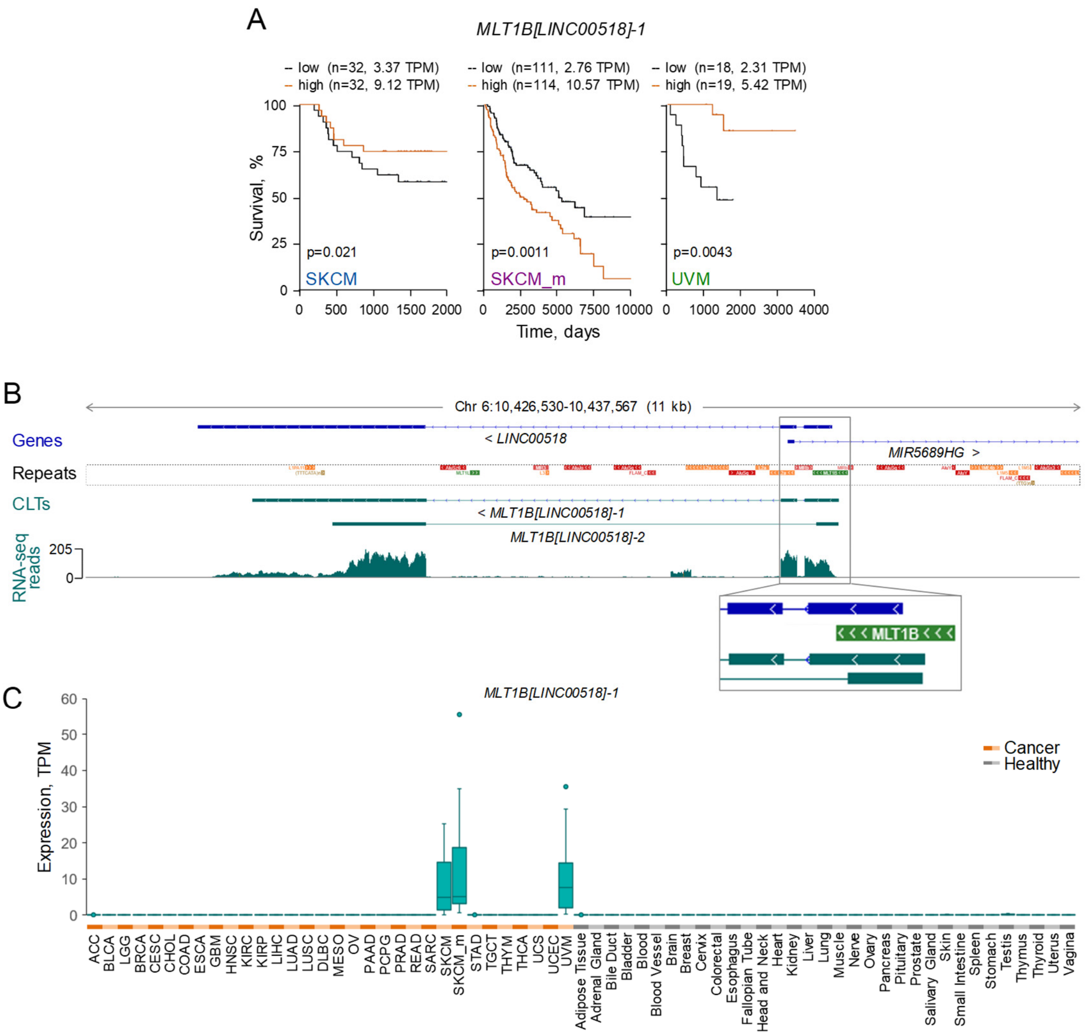

Supplemental Fig S8 Expression and prognostic properties of *MLT1B[LINC00518]-1* in melanoma. (**A**) Kaplan-Meier plots and p-values from log-rank tests for melanoma patients stratified according to the higher versus the lower expression tertiles for *MLT1B[LINC00518]-1*. The number of cases and the expression thresholds are also indicated in brackets. (**B**) GENCODE annotated transcripts at the indicated genomic location (Genes), repeat content (Repeats), structure of two CLTs in the current assembly (CLTs) and RNA-seq trace of a representative SKCM sample. (**C**) Box plot of *MLT1B[LINC00518]-1* expression in cancer patient and healthy control samples.

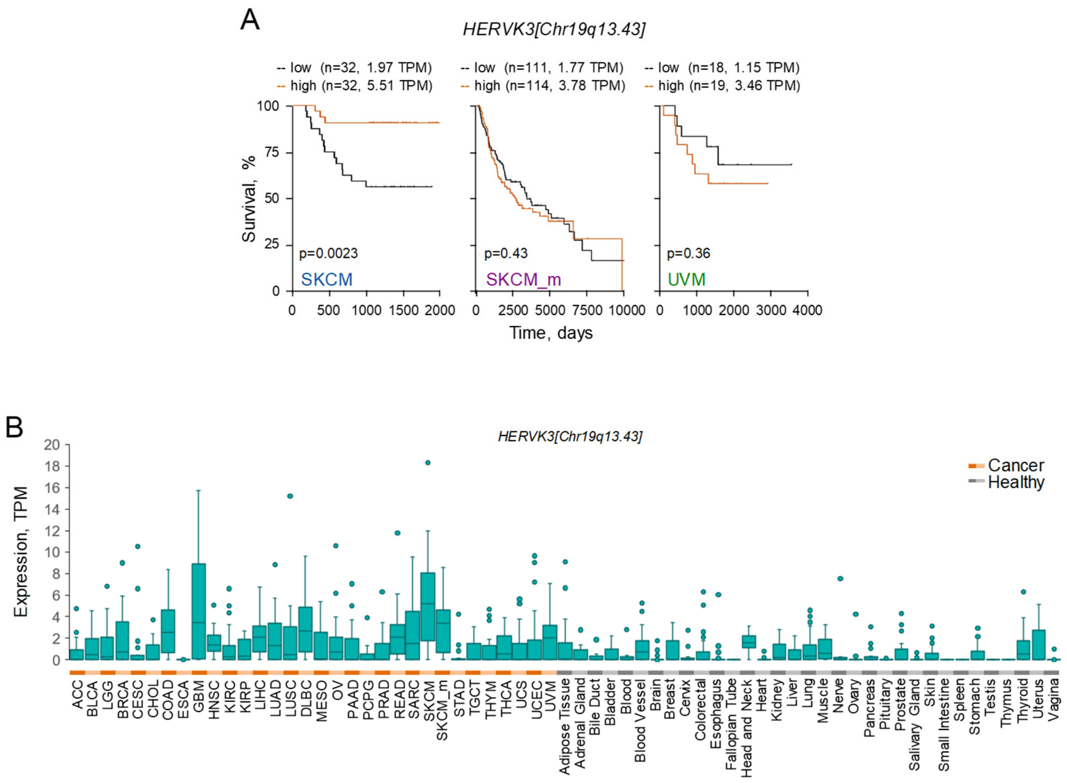

Supplemental Fig S9 Expression and prognostic properties of *HERVK3[Chr19q13.43]* in melanoma. (**A**) Kaplan-Meier plots and p-values from log-rank tests for melanoma patients stratified according to the higher versus the lower expression tertiles for *HERVK3[Chr19q13.43]*. The number of cases and the expression thresholds are also indicated in brackets. (**B**) Box plot of *HERVK3[Chr19q13.43]* expression in cancer patient and healthy control samples.

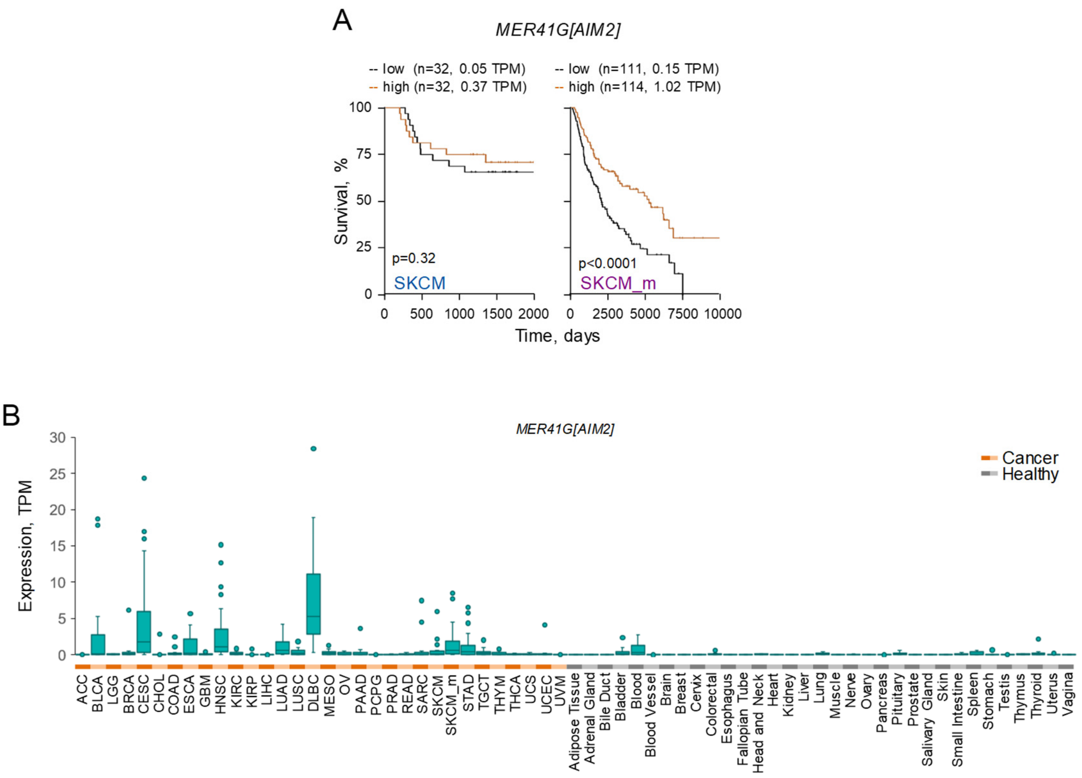

Supplemental Fig S10 Expression and prognostic properties of *MER41G[AIM2]* in melanoma. (**A**) Kaplan-Meier plots and p-values from log-rank tests for SKCM patients stratified according to the higher versus the lower expression tertiles for *MER41G[AIM2]*. The number of cases and the expression thresholds are also indicated in brackets. No association was observed in UVM due to lack of expression. (**B**) Box plot of *MER41G[AIM2]* expression in cancer patient and healthy control samples.

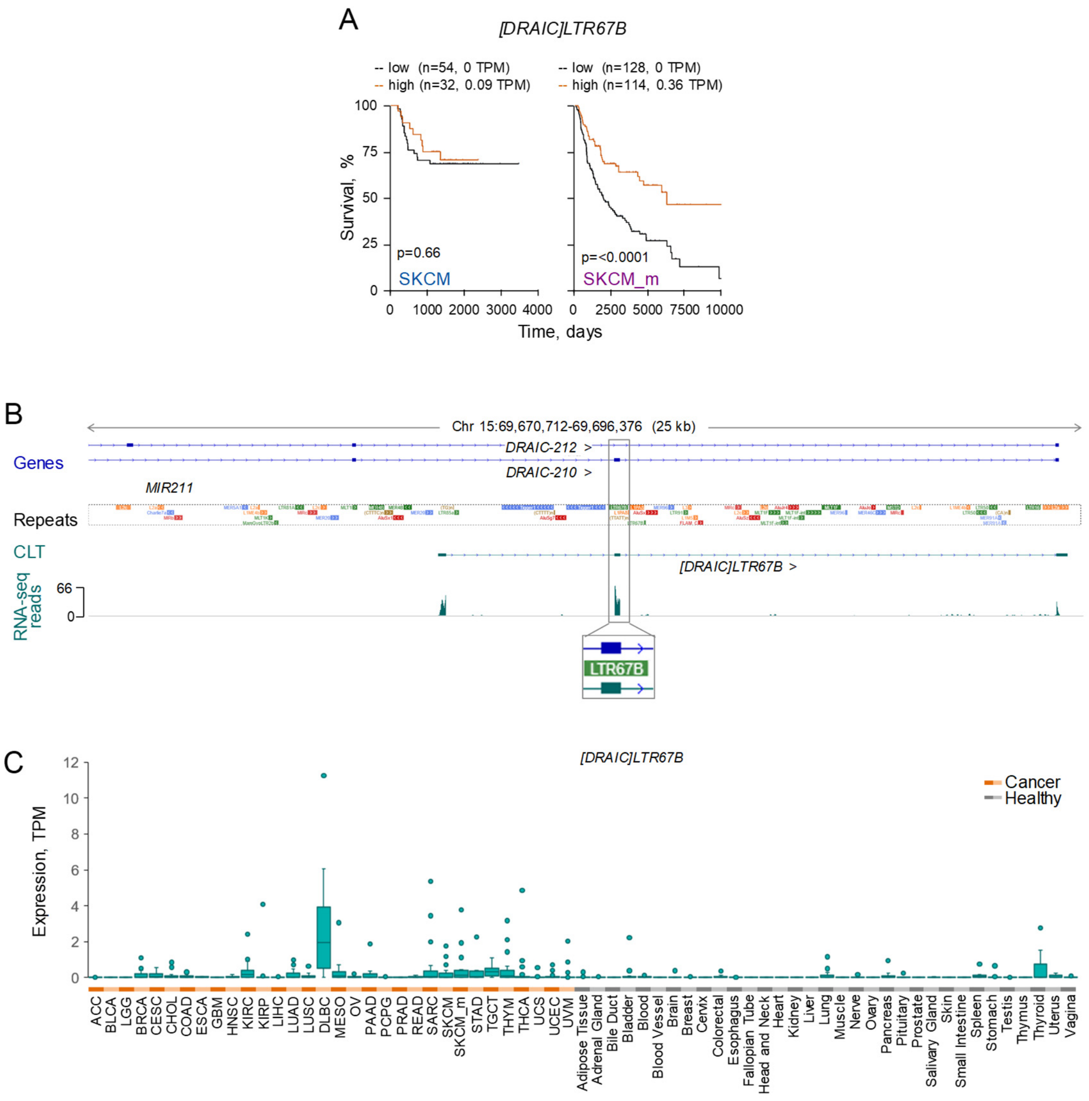

Supplemental Fig S11 Expression and prognostic properties of *[DRAIC]LTR67B* in melanoma. (**A**) Kaplan-Meier plots and p-values from log-rank tests for melanoma patients stratified according to the higher versus the lower expression tertiles for *[DRAIC]LTR67B*. The number of cases and the expression thresholds are also indicated in brackets. No association was observed in UVM due to lack of expression. (**B**) GENCODE annotated transcripts at the indicated genomic location (Genes), repeat content (Repeats), structure of *[DRAIC]LTR67B* in the current assembly (CLT) and RNA-seq trace of a representative SKCM sample. (**C**) Box plot of *[DRAIC]LTR67B* expression in cancer patient and healthy control samples.

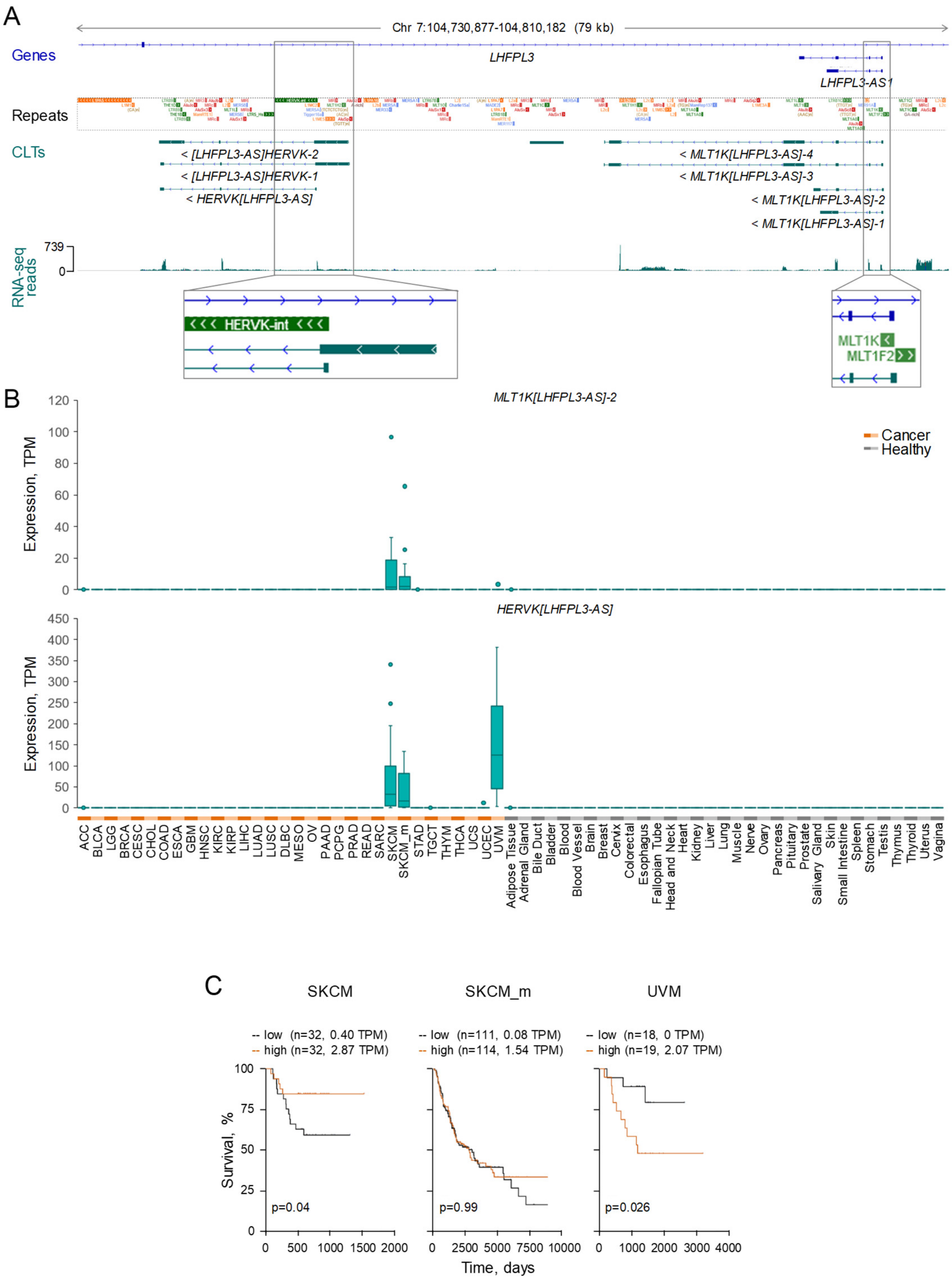

Supplemental Fig S12 Expression and prognostic properties of CLTs at the *LHFPL3* locus in melanoma. (**A**) GENCODE annotated transcripts at the indicated genomic location (Genes), repeat content (Repeats), structure of indicated CLTs at the *LHFPL3* locus in the current assembly (CLTs) and RNA-seq trace of a representative SKCM sample. (**B**) Box plot of *MLT1K[LHFPL3-AS]-2* or *HERVK[LHFPL3-AS]* expression in cancer patient and healthy control samples. (**C**) Kaplan-Meier plots and p-values from log-rank tests for melanoma patients stratified according to the higher versus the lower expression tertiles for *LHFPL3*.

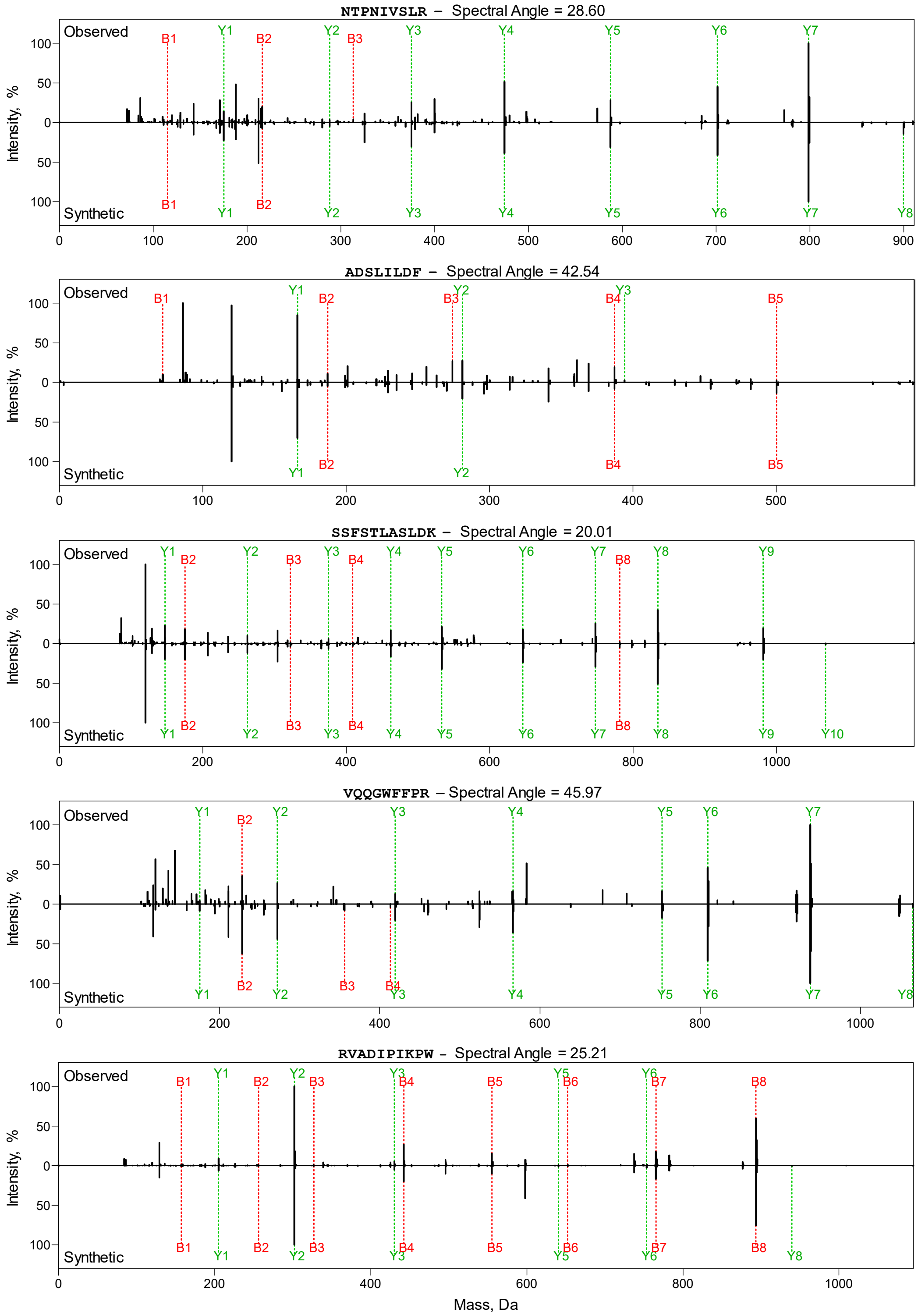

Supplemental Fig S13 Validation of identified peptides with synthetic peptides. Mirror plots of mass spectra corresponding to observed (*top*) and synthetic (*bottom*) peptides of the same sequence. Product ions are also indicated.

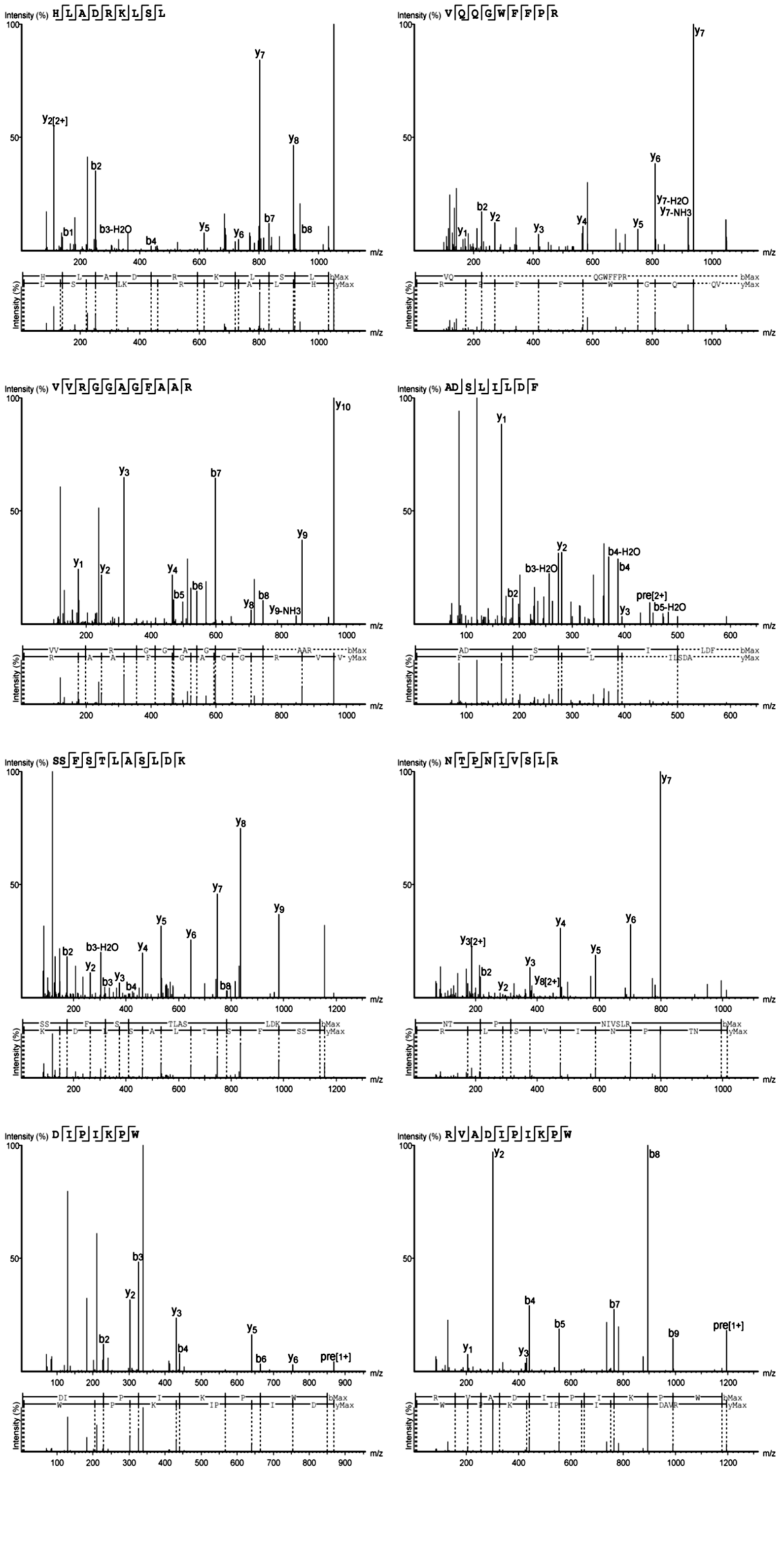

Supplemental Fig S14 Peptide identification with PEAKS. Mass spectra of the indicated CLT-derived peptides, identified in the PXD004894 dataset with PEAKS . Product ions are also indicated.

A

Predicted MHC binder (HLA-A)

Peptide Sequence	HLA-A*01:01	HLA-A*02:02	HLA-A*03:01	HLA-A*24:02	HLA-A*26:01	HLA-A*31:01	HLA-A*33:01	HLA-A*68:01
RVADIPIKP	30,461	22,335	13,416	33,298	30,646	18,578	42,514	18,635
RVADIPIKPW	33,619	22,124	13,164	16,087	27,985	14,233	40,460	25,114
NTPNIVSLR	23,315	31,975	8,425	34,872	6,526	161	54	5
MNTPNIVSL	23,308	15,389	28,657	21,244	23,826	18,979	27,595	20,559
HLADRKLSL	19,894	256	10,621	27,466	14,022	15,616	26,536	29,886
VVRGGAGFAAR	39,751	33,887	8,037	45,045	30,619	243	737	13,768
ARLQGSVT	39,608	43,061	43,368	45,865	39,387	42,844	47,781	47,660
VQQGWFFPR	27,887	12,841	5,583	10,373	24,238	4	56	2,165
SSFSTLASLDK	27,285	22,021	817	39,794	32,736	5,795	19,401	2,192
ADSLILDF	19,176	29,967	35,171	34,074	36,990	39,228	46,365	44,098
ESSAETSP	29,869	45,638	43,440	46,462	29,625	44,487	45,881	40,133
AETSPADSLIL	22,981	34,060	34,273	36,430	29,375	39,035	42,678	29,890

Predicted MHC binder (HLA-B)

Peptide Sequence	HLA-B*07:02	HLA-B*08:01	HLA-B*15:01	HLA-B*27:05	HLA-B*39:01	HLA-B*40:01	HLA-B*58:01
RVADIPIKP	26,033	33,916	24,696	20,377	31,565	21,822	14,810
RVADIPIKPW	8,804	27,610	3,310	18,779	32,033	31,758	178
NTPNIVSLR	31,410	30,147	38,132	24,787	35,288	32,155	30,751
MNTPNIVSL	11,597	3,896	11,167	20,245	896	11,275	9,242
HLADRKLSL	458	42	1,392	12,559	1,871	20,639	18,477
VVRGGAGFAAR	16,152	34,329	31,049	11,814	37,753	37,146	38,335
ARLQGSVT	26,308	28,228	36,906	10,770	33,038	39,309	38,987
VQQGWFFPR	28,336	28,607	5,305	494	7,029	12,250	26,259
SSFSTLASLDK	32,884	34,931	30,390	30,982	25,958	36,015	22,577
ADSLILDF	38,628	40,127	29,585	37,439	39,094	32,271	26,262
ESSAETSP	41,715	38,906	32,910	41,270	39,410	26,813	23,652
AETSPADSLIL	36,138	25,183	32,775	36,858	30,467	266	18,748

Strong
Weak
Non-binder

Predicted MHC binder (HLA-C)

Peptide Sequence	HLA-C*03:03	HLA-C*04:01	HLA-C*05:01	HLA-C*06:02	HLA-C*07:01	HLA-C*07:02	HLA-C*08:02	HLA-C*12:03	HLA-C*14:02	HLA-C*15:02
RVADIPIKP	20,352	22,266	22,364	36,596	31,532	35,176	33,533	6,164	36,593	10,092
RVADIPIKPW	24,372	38,809	27,344	46,681	45,320	40,855	32,317	16,299	38,190	39,338
NTPNIVSLR	43,315	11,977	29,464	25,314	20,987	39,581	30,638	27,017	16,357	30,749
MNTPNIVSL	91	10,452	28,443	8,166	1,682	2,350	22,412	83	718	12,640
HLADRKLSL	245	9,358	31,180	6,853	2,635	3,349	19,532	1,185	3,005	7,452
VVRGGAGFAAR	47,078	38,123	47,245	45,702	45,714	44,129	45,523	44,280	45,133	47,117
ARLQGSVT	47,072	42,625	47,280	32,156	36,488	38,481	44,894	45,057	45,829	47,073
VQQGWFFPR	28,222	10,843	43,156	45,699	41,759	18,323	32,628	25,324	22,146	29,645
SSFSTLASLDK	36,483	34,472	47,207	39,985	38,371	43,466	42,155	38,297	34,289	31,588
ADSLILDF	45,130	38,316	12,335	45,724	44,150	42,543	24,798	40,992	45,920	45,271
ESSAETSP	46,300	43,559	47,563	47,842	47,024	47,492	42,103	45,850	45,898	42,032
AETSPADSLIL	35,632	37,550	44,507	42,836	41,223	43,202	39,206	40,932	36,846	20,363

B

Melanoma Patient HLA type	Identified peptide in respective patient: Score	% Rank
**Mel5**	**RVADIPIKPW**	
HLA-A*01	0.015	7.231
HLA-A*25	0.066	0.999
HLA-B*08	0.003	21.977
HLA-B*18	0.003	8.546
**Mel12**	**NTPNIVSLR**	
HLA-A*01:01	0.038	4.199
HLA-B*08:01	0.001	36.707
HLA-C*07:01	0.011	8.543
**Mel15**	**NTPNIVSLR**	
HLA-A*03:01	0.059	2.734
HLA-A*68:01	0.924	0.015
HLA-B*27:05	0.001	15.182
HLA-B*35:03	0.000	34.200
HLA-C*02:02	0.023	10.541
HLA-C*04:01	0.008	9.707
**Mel16**	**HLADRKLSL**	
HLA-A*01:01	0.026	5.164
HLA-A*24:01	0.016	5.838
HLA-B*07:01	0.228	0.743
HLA-B*08:01	0.893	0.009
HLA-C*07:01	0.321	0.300
HLA-C*07:02	0.429	0.390

Strong
Weak
Non-binder

Supplemental Fig S15 Predicted MHC class I binding affinities of identified peptides. (**A**) Affinities of the eluted CLT peptides for the indicated major HLA allotypes, as calculated using NetMHC4.0. Peptides with strong or weak binding affinity are also indicated by different colors. (**B**) Scores and % Ranks of peptides eluted from a given patient for binding to the HLA alleles of that patient, as calculated using NetMHCpan4.0. Only patients from whom CLT-derived peptides were eluted and the HLA haplotype was known are included (Mel5, Mel12, Mel15 and Mel16, according to Bassani-Sternberg et al. 2016).

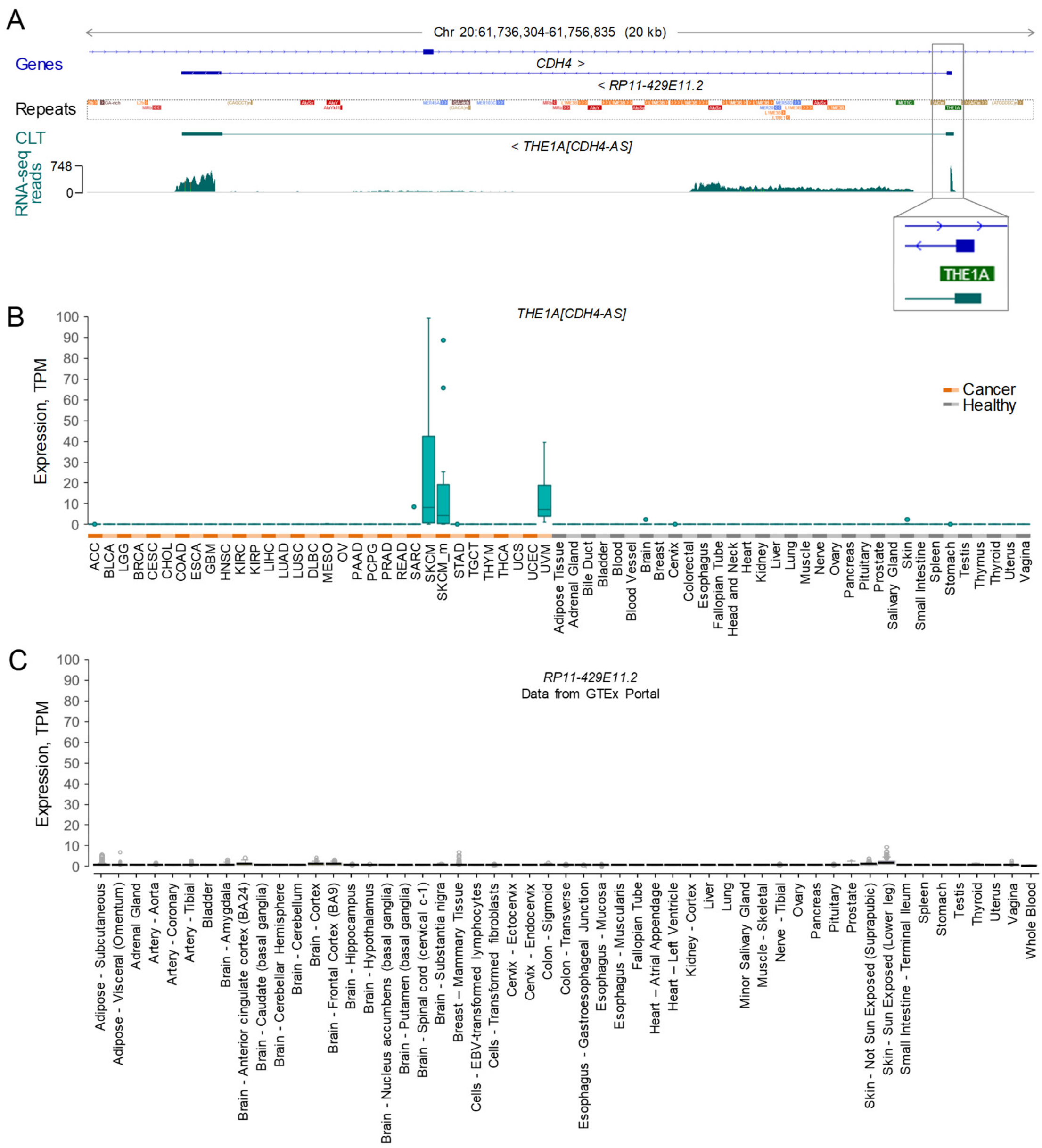

Supplemental Fig S16 Structure and expression of *THE1A[CDH4-AS]* in melanoma. (**A**) GENCODE annotated transcripts at the indicated genomic location (Genes), repeat content (Repeats), structure of *THE1A[CDH4-AS]* in the current assembly (CLT) and RNA-seq trace of a representative SKCM sample. (**B**) Box plot of *THE1A[CDH4-AS]* expression in cancer patient and healthy control samples. (**C**) Box plot of *RP11-429E11.2* expression in a collection of healthy control samples from GTEx Portal. Although the scale of the y-axes in (**B**) and (**C**) were plotted to the same scale, the larger transcriptome that we use as denominator in our assembly produces lower overall TPM values than estimated on GTEX Portal.

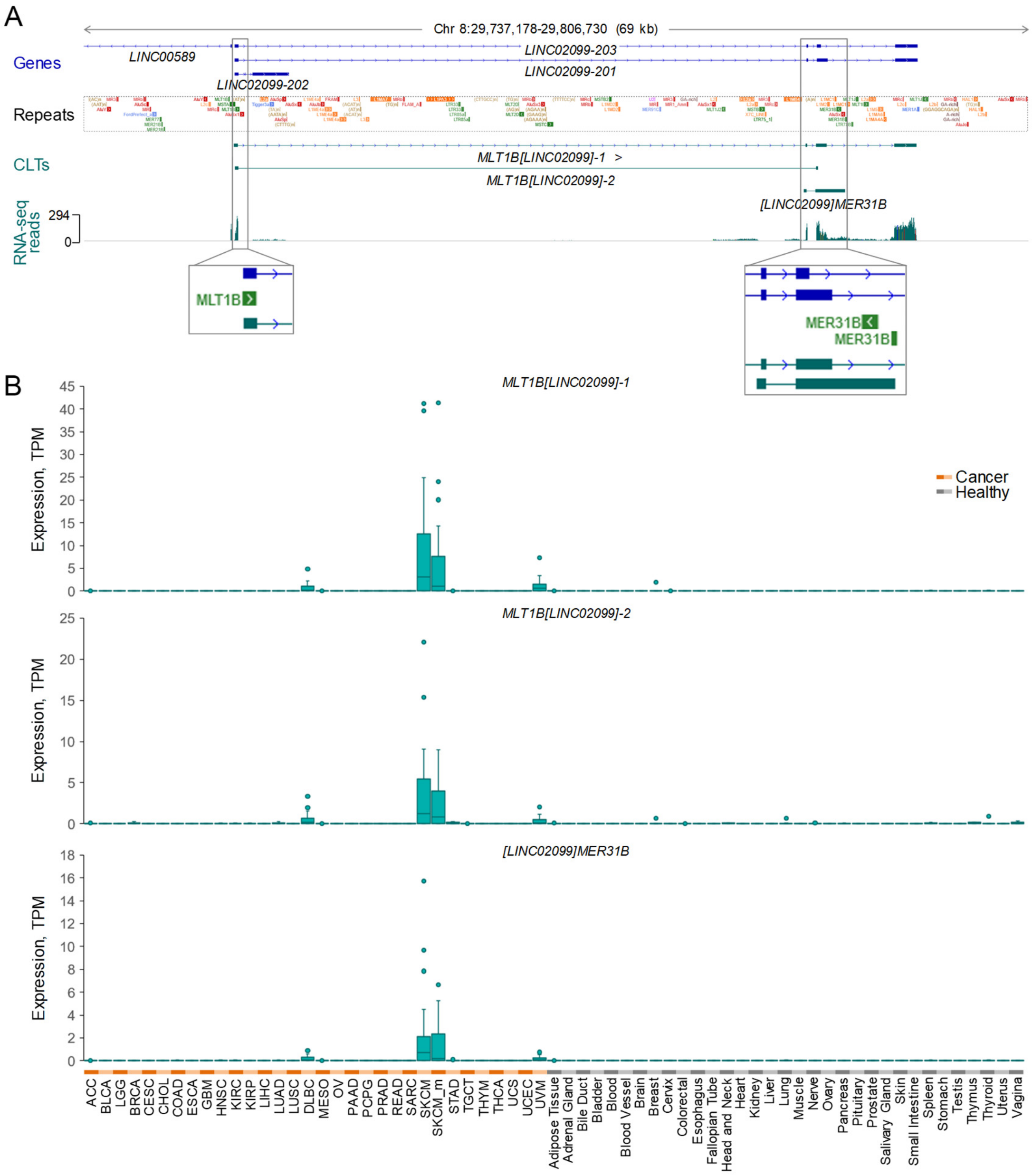

Supplemental Fig S17 Structure and expression of CLTs at the *LINC02099* locus in melanoma. (**A**) GENCODE annotated transcripts at the indicated genomic location (Genes), repeat content (Repeats), structure of indicated CLTs at the *LINC02099* locus in the current assembly (CLTs) and RNA-seq trace of a representative SKCM sample. (**B**) Box plot of *MLT1B[LINC02099]-1*, *MLT1B[LINC02099]-1* or *[LINC02099]MER31B* expression in cancer patient and healthy control samples.
