# Supplementary material for: LTR retroelement expansion of the human cancer transcriptome and immunopeptidome revealed by de novo transcript assembly
Source: Genome Res. 2019 Oct;29(10):1578–90. doi: 10.1101/gr.248922.119 (PMC6771403; doi:10.1101/gr.248922.119)
Supplement: Supplemental Material [file supp_gr.248922.119_Supplemental_Code_S1.docx]

##### This code was used to estimate expression of all Trinity assembled transcripts using quasi-mapping of Salmon in samples from TCGA or GTEx. Executable lines are marked with ‘>’. The bash script assumes the Salmon transcriptome index was generated prior to executing and is provided as first Argument. The bam or fastq file name is provided as second Argument.

> salmon index –t flattened_transcriptome.fasta -i ./transcriptome_index

Salmon version 0.8.2

SAMtools version 1.3.1

Python version 3.5.2

TCGA paired-end RNA-seq was downloaded through GDC portal as bam files. GTEx paired-end RNA-seq was downloaded with the SRA toolkit as fastq files. Hence, TCGA files were first converted to fastq files. Here, the following programs were used from [github.com/A-N-Other/pedestal:](http://github.com/A-N-Other/pedestal:) deinterleave (commit 169dd81) and interleavei (commit 800e78f). They aare available under the permissive MIT license and we encourage code re-use and comment.

#########################

##### Step 1

### file input & directory and test if file exists

> indexfile=$1

> bamname=$2 #collects string of input file from 1st argument (*.bam)

> fullfilename="${bamname##*/}"

> indirectory="${bamname%/*}"

> subdirectory="${indirectory##*/}"

> filename="${fullfilename%_gdc_realn_rehead.bam}"

#> filename="${fullfilename%_1.fastq.gz}" # for GTEx fastq files

> if [ -s ./countFiles/"$subdirectory"/"$filename".counts.gz ]; then

exit 3; fi

#########################

#########################

##### Step 2

### separate first and second read of every pair with deinterleave script

> samtools fastq -n "$bamname" | interleave -u -

| deinterleave ~/Scratch/tcgarecycle/fastq.files/"$filename"_1.fq ~/Scratch/tcgarecycle/fastq.files/"$filename"_2.fq

#########################

#########################

##### Step 3

### Expression estimate with Salmon quasi-mapping

> ~/bin/salmon-0.8.2/bin/salmon quant \

-p 8 -i "$indexfile" \

-l IU --seqBias --gcBias \

-1 ~/Scratch/tcgarecycle/fastq.files/"$filename"_1.fq \

-2 ~/Scratch/tcgarecycle/fastq.files/"$filename"_2.fq \

-o ./countFiles/"$subdirectory"/"$filename".tmpdirectory

> mv ./countFiles/"$subdirectory"/"$filename".tmpdirectory/quant.sf \ ./countFiles/"$subdirectory"/"$filename".counts

> mv ./countFiles/"$subdirectory"/"$filename".tmpdirectory/aux_info/ambig_info.tsv \ ./countFiles/"$subdirectory"/"$filename".ambig_info.tsv
