# Supplementary material for: LTR retroelement expansion of the human cancer transcriptome and immunopeptidome revealed by de novo transcript assembly
Source: Genome Res. 2019 Oct;29(10):1578–90. doi: 10.1101/gr.248922.119 (PMC6771403; doi:10.1101/gr.248922.119)
Supplement: Supplemental Material [file supp_gr.248922.119_Supplemental_Code_S2.R.html]

Supplemental\_Code\_S2 

# LTR retroelement expansion of the human cancer transcriptome and immunopeptidome revealed by de novo transcript assembly
